# Merlin Isoforms 1 and 2 Both Act as Tumour Suppressors and Are Required for Optimal Sperm Maturation

**DOI:** 10.1371/journal.pone.0129151

**Published:** 2015-08-10

**Authors:** Ansgar Zoch, Steffen Mayerl, Alexander Schulz, Thomas Greither, Lucien Frappart, Juliane Rübsam, Heike Heuer, Marco Giovannini, Helen Morrison

**Affiliations:** 1 Leibniz Institute for Age Research—FLI Jena, Beutenbergstr. 11, D-07745 Jena, Germany; 2 Center for Reproductive Medicine and Andrology, Martin-Luther-University Halle-Wittenberg, Ernst-Grube-Str. 40, D-06120 Halle (Saale), Germany; 3 Leibniz Research Institute for Environmental Medicine, Auf’m Hennekamp 50, D-40225 Düsseldorf, Germany; 4 Center for Neural Tumor Research and Section on Genetics of Hereditary Ear Disorders, House Research Institute, University of California Los Angeles, Los Angeles, CA 90057, United States of America; University Hospital of Münster, GERMANY

## Abstract

The tumour suppressor Merlin, encoded by the gene *NF2*, is frequently mutated in the autosomal dominant disorder neurofibromatosis type II, characterised primarily by the development of schwannoma and other glial cell tumours. However, *NF2* is expressed in virtually all analysed human and rodent organs, and its deletion in mice causes early embryonic lethality. Additionally, *NF2* encodes for two major isoforms of Merlin of unknown functionality. Specifically, the tumour suppressor potential of isoform 2 remains controversial. In this study, we used *Nf2* isoform-specific knockout mouse models to analyse the function of each isoform during development and organ homeostasis. We found that both isoforms carry full tumour suppressor functionality and can completely compensate the loss of the other isoform during development and in most adult organs. Surprisingly, we discovered that spermatogenesis is strictly dependent on the presence of both isoforms. While the testis primarily expresses isoform 1, we noticed an enrichment of isoform 2 in spermatogonial stem cells. Deletion of either isoform was found to cause decreased sperm quality as observed by maturation defects and head/midpiece abnormalities. These defects led to impaired sperm functionality as assessed by decreased sperm capacitation. Thus, we describe spermatogenesis as a new *Nf2*-dependent process. Additionally, we provide for the first time *in vivo* evidence for equal tumour suppressor potentials of Merlin isoform 1 and isoform 2.

## Introduction

The neurofibromatosis type II (*NF2*) gene encodes the tumour suppressor protein Merlin, which belongs to the ezrin-radixin-moesin (ERM) family of actin-binding proteins. Functionally, Merlin is unique within this family, being the only tumour suppressor. Loss of Merlin due to germline *NF2* gene mutations leads to neurofibromatosis type 2 (NF2) disease, a nervous system cancer syndrome [1] where patients primarily develop schwannomas and meningiomas. Furthermore, somatic *NF2* mutations have also been reported in a large proportion of sporadic peripheral and central nervous system tumours [2].

Studies on the expression pattern of *NF2*/*Nf2* in humans, rats and mouse embryos have shown that it is expressed in virtually all analysed organs [3–7]. Although the hallmark of NF2 disease is glial cell tumours, there is increasing evidence that the ubiquitously expressed Merlin protein functions as a tumour suppressor in multiple cell types, including, for example, breast carcinoma and melanoma [6, 8]. In addition, *in vivo* models involving the deletion of Merlin have facilitated not only the understanding of Merlin’s function as a tumour suppressor in multiple cell types but also the elucidation of a critical role for Merlin in development [9, 10].

Heterozygous *Nf2* knockout mouse models develop a variety of cancers, including hepatomas and osteosarcomas [10]. In addition, liver-specific deletion of Merlin results in liver overgrowth and tumourigenesis [11] [12] [13]. Interestingly, Merlin loss in the skin shows no effect on progenitor proliferation but instead leads to loss of progenitor cell polarity via an alteration of adhesion junctions [14]. Moreover, in bone tissue, conditional deletion of both *Nf2* alleles has revealed a role for Merlin in the regulation of the haematopoietic stem cell microenvironment [15]. Finally, we have recently shown that Merlin plays a neuron-intrinsic role in the regulation of dendrite formation and axon calibre control in the central and peripheral nervous systems [16, 17]. Collectively, these studies demonstrate not only Merlin’s broad function but also its cell type-specific mechanism of action.

The human gene *NF2* and its close murine homologue *Nf2* encode for 17 exons that are subject to alternative splicing [18]. By far the most abundant isoforms are isoform 1 and isoform 2 (sometimes denoted as isoform 3), which comprise the full-length isoforms [5]. Exon-skipping of exon 16 leads to the production of isoform 1 mRNA that encodes 16 amino acids (LTLQSAKSRVAFFEEL) specific to Merlin isoform 1. Retention of exon 16 leads to translation of an altered C-terminus with 11 unique amino acids (PQAQGRRPICI) in Merlin isoform 2 [5].

The specific function of isoform 1 and 2 is controversial. Of the two major Merlin isoforms, only isoform 1 was originally thought to have tumour suppressor potential [19–21]. However, more recent studies suggest that both isoforms have relatively equal growth suppressive potentials and act similarly *in vitro* [22–26].

We recently reported the first specific function of *Nf2* isoform 2 *in vivo*, demonstrating that axon calibre regulation depends on isoform 2 in neurons. We found that mice deficient specifically in *Nf2* isoform 2 develop polyneuropathies similar to patients with NF2 disease [17]. Our discovery of an isoform-specific function in neurons raises the possibility of functional specialisation of the two major *Nf2* isoforms. To the best of our knowledge, no pathogenic mutation that specifically strikes one isoform of *NF2* has been described to date–tumourigenic mutations always inactivate both isoforms [27, 28]. Therefore, NF2 syndromes do not offer an insight into the specific functions of Merlin isoform 1 and isoform 2. Since positive evolutionary selection pressure must be exerted on the maintenance of two different C-termini [18], it is likely that they perform distinct functions in some cells or at some point during development.

To investigate this notion further, the present study examined the *in vivo* significance of the two major Merlin isoforms by employing mouse models deficient in either *Nf2* isoform 1 or isoform 2. Specifically, we aimed to clarify the tumour suppressor potential of both isoforms and analyse the effect of isoform inversion in organs physiologically expressing primarily one isoform. In this study we show that deletion of either *Nf2* isoform does not cause tumour formation in mice but unexpectantly decreases sperm quality, demonstrating a novel role for Merlin in spermatogenesis.

## Materials and Methods

### Animal housing and genotyping

Animals were kept according to local governmental and institutional animal care regulations (Zweckverband Veterinär- und Lebensmittelüberwachungsamt Jena-Saale-Holzland). Animal experiments were approved and licensed by the same authorities (see above; license number: “J-SHK-2684-04-08-02/14"). Mice were maintained under a 12-h light/12-h dark cycle at 22°C and provided with standard laboratory chow and tap water *ad libitum*. Mice were culled by cervical dislocation or CO_2_ asphyxiation at the stated age. To prevent suffering, ageing mice were sacrificed upon signs of injury (e.g. scratch wound) or illness (e.g. tumour development). The *Nf2* iso1 ko and iso2 ko animals–described in [17]–were backcrossed at least six times into a C57Bl/6 background from an FVB/NJ-C57Bl/6 background.

Tail clip lysates were used to genotype the mouse lines. Genotyping of iso1 ko and iso2 ko animals was conducted using the iso1 ko and iso2 ko primer pairs (Table 1), respectively, yielding a wildtype (product size of iso1 ko: 267 bp, iso2 ko: 378 bp) and a knockout (product size of iso1 ko: 391 bp, iso2 ko: 235 bp) PCR product.

**Table 1:** 

Genotyping primer	Primer 1 [5’–3’]	Primer 2 [5’–3’]
Iso1 ko	CCTCAAGCCCAAGGCAGAAGA	CTTCAGAGTGAGGCAGTCTTCTAGG
Iso2 ko	CAGTACACCTGAGGTCACTGTCTC	CTTCAGAGTGAGGCAGTCTTCTAGG

Animals of different ages were analysed. Young mice are denoted according to age in days as P8, P18 or P35 (post-natal day). Adult animals are denoted as young (2–3 months old) or old (16–18 months old) if age is not specified.

### Organ sample preparation and storage

Organ samples were obtained from animals killed by cervical dislocation. For histological preparations, liver and skeletal muscle tissue was fixed overnight at 4°C in phosphate-buffered saline (PBS)-buffered 4% paraformaldehyde (PFA), pH 7.0. Testis tissue was fixed in Bouin’s fluid (0.9% picric acid, 10% formaldehyde, 5% glacial acetic acid) overnight and then washed multiple times in 70% ethanol. Following fixation, tissue was embedded in paraffin.

Testis used for *in situ* hybridisation (ISH) was frozen in isopentane on dry ice and then stored at –80°C. Cryosections of 20 μm thickness were cut for the ISH procedure on a cryostat (Leica CM 3050S) at –20°C, thaw mounted onto silane-treated slides (Superfrost Plus; Thermo Scientific), air dried and stored at –80°C.

For mRNA extraction and protein lysate preparation, organs were snap frozen in liquid nitrogen and stored at –80°C.

### Histological stainings

Paraffin blocks were sliced to generate 5-μm thick sections which were dewaxed and rehydrated using a graded alcohol series and then stained with H&E (haematoxylin & eosin), PAS (periodic acid-Schiff) or Masson’ trichrome stains according to standard protocols [29]. Stained tissues were embedded in xylene based medium under a coverslip and analysed by light microscopy using an Olympus AX70 microscope. Picture quality was optimized by Photoshop (Adobe).

### Immuno histo chemisty (IHC)

IHC was performed on paraffin section using the Golden Bridge International Polink-2 Plus HRP rabbit kit with AEC chromogen (cat. No. D40-15) according to the manufacturer’s recommendations. Antigen retrieval was performed by heating the slides to 100°C in 10 mM citrate-buffer for 10 min using a pressure cooker. The Merlin C-18 antibody (Santa Cruz Biotechnology #sc-332) was diluted 1:200.

### In situ hybridisation (ISH)

A cDNA fragment corresponding to nt 497–997 of mouse *Nf2* cDNA (transcript ID NM 001252250.1) recognising exons 1–4 as a total *Nf2* transcript probe was generated by PCR using the ISH-mmNf2-ex2 primer pair (Table 2) and subcloned into the pCR2-TOPO vector (Invitrogen). Radiolabelled riboprobes were generated using [^35^S]UTP (Hartmann Analytic GmbH) as substrate for the *in vitro* transcription reaction and T7 as well as SP6 polymerases for preparing antisense and sense riboprobes, respectively, according to standard protocols.

The detailed ISH procedure is described in the supplementary methods. Experiments conducted with the respective sense probes did not produce any hybridisation signals.

**Table 2:** 

ISH primer	Primer 1 [5’–3’]	Primer 2 [5’–3’]
ISH-mmNf2-ex2	GGGGCTAAGAGACCCAGAAC	GACAGCATATGACGCCAAGA

### RT-PCR and qPCR

mRNA was isolated from liquid nitrogen snap-frozen organs with Qiagen’s RNeasy kit (#75142) according to the instructions using DNase (#79254, Qiagen) on-column digestion, Trizol (Invitrogen) pre-lysis and mRNA extraction using the Precellys homogenisation system (Peqlab) with zirconium beads. For small sample sizes and cells, Macherey-Nagel’s NucleoSpin RNA XS kit (#740902) was used. cDNA was produced using the Omniscript kit from Qiagen (#205111). RT-PCR was performed using MyTaq 2× Mastermix (#BIO-25043, Bioline) and 0.5 μM primer concentration (Table 3). qPCR (quantitative real-time PCR) was performed using Invitrogen’s Fast SYBR Green Master Mix (#4385616) on a Roche Lightcycler 480. In order to calculate relative mRNA levels, one sample was serially diluted to produce a 4x, 2x, 1x, 0.5x and 0.25x standard. Amplification efficiency was calculated for each primer pair using these standards and the second derivative maximum of the fluorescence intensity curve as a cycle call. mRNA levels of all other samples were then calculated according to the standard curve and therefore describe relative abundance to the sample used as a standard (in figures this is referred to as “normalized to X”, where x is the standard sample).

**Table 3:** 

qPCR primer	Primer 1 [5’–3’]	Primer 2 [5’–3’]
Nf2ex14-17 (RT-PCR both *Nf2* isoforms)	AGCTTCGACATTATTGCTGACAG	TCAGGAGCAGCAGATGTGGCAG
Nf2ex1-2 (qPCR total *Nf2*)	AAGCAGCCCAAGACATTCAC	AAGAACCAGGTTTCCCGAAG
Nf2ex14-15/17 (qPCR *Nf2* isoform 1)	ACGGACATGAAGCGACTTTC	GCTCTGCAGAGTGAGCTTTTTAAT
Nf2ex14-16 (qPCR *Nf2* isoform 2)	ACGGACATGAAGCGACTTTC	GCAGATAGGTCTTCTGCCTTG
Cyclophilin D (*Ppid*) (qPCR)	GCAAGGATGGCAAGGATTGA	AGCAATTCTGCCTGGATAGC
β-Actin (*Actb*) (qPCR)	AGAGGGAAATCGTGCGTGAC	CAATAGTGATGACCTGGCCGT
Zbtb16 (qPCR)	CCACCTTCGCTCACATACAG	GTGGCAGAGTTTGCACTCAA
**qPCR primer**	**Primer 1 [5’–3’]**	**Primer 2 [5’–3’]**
Nf2ex14-17 (RT-PCR both *Nf2* isoforms)	AGCTTCGACATTATTGCTGACAG	TCAGGAGCAGCAGATGTGGCAG
Nf2ex1-2 (qPCR total *Nf2*)	AAGCAGCCCAAGACATTCAC	AAGAACCAGGTTTCCCGAAG
Nf2ex14-15/17 (qPCR *Nf2* isoform 1)	ACGGACATGAAGCGACTTTC	GCTCTGCAGAGTGAGCTTTTTAAT
Nf2ex14-16 (qPCR *Nf2* isoform 2)	ACGGACATGAAGCGACTTTC	GCAGATAGGTCTTCTGCCTTG
Cyclophilin D (*Ppid*) (qPCR)	GCAAGGATGGCAAGGATTGA	AGCAATTCTGCCTGGATAGC
β-Actin (*Actb*) (qPCR)	AGAGGGAAATCGTGCGTGAC	CAATAGTGATGACCTGGCCGT
Zbtb16 (qPCR)	CCACCTTCGCTCACATACAG	GTGGCAGAGTTTGCACTCAA

### Western blotting

Western blot analysis was performed according to standard protocols. The following primary antibodies were incubated overnight at 4°C: Abcam (NF2/Merlin isoform 1 #3357 1:2000), Santa Cruz Biotechnology (Erk 1 K-23 #sc-94 1:1000, Erk 2 K-23 #sc-153 1:1000, GAPDH H-12 #sc-66574 1:1000), Sigma (β-Actin AC-15 #A1978 1:80000), Cell Signaling Technology (phospho Yap #4911 1:1000, Yap #4912 1:500, phospho Erk 1/2 #4370 1:1000, total (N-terminal) Merlin D1D8 #6995 1:1000) and Millipore (phospho-Tyr 4G10 #05-1050X 1:1000). After washing, the blot was incubated with horseradish peroxidase-conjugated anti-rabbit or anti-mouse secondary goat antibodies (Dako) for 1 h at room temperature. Following intensive washing, the horseradish peroxidase signal was developed using ECL (Pierce-Thermo Scientific) and film (Hyperfilm ECL; GE Healthcare).

Signal intensity quantification was carried out on scanned film using ImageJ software (open source software available at rsb.info.nih.gov/ij/).

### Fertility and sperm analysis

Assessment of fertility was carried out by mating one male with one female at the age of 2 months and documenting the litter frequency and size for 6 months.

For sperm analysis, mice were killed and about 3 cm of the seminal duct (vas deferens) was dissected. Sperm were squeezed out with fine forceps. The sperm were taken up in 500 μl Whitten’s medium (non-capacitating medium: 100 mM NaCl, 4.4 mM KCl, 1.2 mM KH_2_PO_4_, 1.2 mM MgSO_4_, 5.4 mM glucose, 0.8 mM pyruvic acid, 4.8 mM lactic acid, 2.4 mM CaCl_2_, 20 mM HEPES (acid); all Sigma-Aldrich) and analysed manually in a Makler chamber according to WHO guidelines [30].

Sperm motility was assessed by manually classifying sperm into three categories: progressive motility (sperm moving in one direction or around in large circles), non-progressive motility (sperm tail is moving but sperm does not cover distance while moving, either being stuck in one place or moving in small circles) or immobility (no movement of sperm or sperm tail).

Sperm count and morphology were assessed from immobilised sperm preparations in 0.1% PFA. Iso1 ko and iso2 ko sperm without cytoplasmic droplets (CD) and head-midpiece-agglutinations (AG) were counted as normal.

Capacitation assay was performed by incubating minced cauda epididymis for 20 min at 37°C in non-capacitating Whitten’s medium to let sperm swim out (swim-up sperm). Swim-up sperm were counted using an improved Neubauer counting chamber. Sperm were then filtered through a 70-μm nylon gauze and incubated for 1 h in capacitating Whitten’s medium (non-capacitating medium supplemented with 15 mM NaHCO_3_ and 5 mg/ml BSA (bovine serum albumin); both Sigma-Aldrich). Non-capacitated and capacitated sperm were washed with PBS, centrifuged at 2000 g for 1 min and the pellet snap frozen in liquid nitrogen.

### Electron microscopy

Swim-up sperm was promptly fixed in Karnovsky fixative (2% PFA, 2.5% glutaraldehyde (Roth) in PBS, pH 7.3) for 12 h. Sperm were then embedded in 3% agar (Roth). Samples were processed on a tissue processor EMTP (Leica). Samples were washed with PBS, fixed again with 1% osmic acid (Roth) and 1% potassium hexacyanoferrate (Roth) in PBS for 2.5 h at 4°C. Samples were then dehydrated using increasing concentrations of acetone and embedded in epon (glycid ether 100; Serva) in gelatine capsules. Epon was allowed to polymerise for 48 h at 60°C. Ultra-thin sections were cut (Reichert Ultracut S (Leica) and diamond blade 35° (Diatome)) with further contrasting onto copper grids. Sections were analysed with a JEM 1400 (JEOL) with an accelerating voltage of 80 kV.

### Fluorescence-activated cell sorting (FACS)

FACS of spermatogonial stem cells was performed according to the method described by Takubo et al. [31]. Mice were sacrificed and testes dissected and placed in warm PBS. The tunica albuginea was removed and the testicular cords pre-digested for 20 min with 0.1% collagenase at 37°C. Another round of collagenase digestion for 30 min was followed by digestion with 1× Trypsin-EDTA solution (PAA) and subsequent filtering through a 40-μm nylon mesh. The single cell solution was then incubated with an antibody against EpCam (clone G8.8 1:100; #14–5791, eBioscience) and MACS (magnet-activated cell sorting) sorted using the autoMACS system from Miltenyi Biotec GmbH. The positive cells were prepared for FACS by incubation with anti-Integrin α6-PE (clone eBioGoH3 1:100; #12–0495, eBioscience) and anit-c-Kit-APC (clone 2B8 1:100; #17–1171, eBioscience). The sorting was performed on a BD Biosciences FACS Aria 2. Viable cells were gated according to DAPI (Sigma-Aldrich) staining and forward scatter. Cell fractions were sorted according to c-Kit and Integrin α6 staining intensity.

### Oil Red O staining

Oil Red O staining was performed on cryosections according to standard procedures [29]. Briefly, samples were incubated in Oil Red O solution for 20 min, washed with water, counterstained with haematoxylin and embedded under coverslips in aqueous mounting medium.

### Luciferase assays

Luciferase assays were performed using the Promega Dual Glo Luciferase assay kit according to the manufacturer‘s instructions. Cells were transfected with a firefly luciferase reporter plasmid containing the CTGF promoter [32] and the pRL-TK (Promega) vector containing the renilla luciferase under the control of the thymidine kinase promoter using Lipofectamine 2000 (Invitrogen). For overexpression of merlin, pcDNA3-based *Nf2* full-length sequences for isoform 1 and 2 was used. The empty backbone vector pcDNA3 was used for the control transfection. Merlin c-terminal fragments consisted of amino acids 300–595 (iso1 c-term) and 300–590 (iso2 c-term).

### Hanging wire test

The “hanging wire” test for muscle strength was performed as follows: Mice were put onto a metal grid, which was then turned upside down. Precautions were taken so that the mice could not climb up the side of the grid. The time until mice fell off the grid was measured or the experiment was terminated after 60 s.

### Statistical analysis

Quantitative data were analysed using the software R (open source software available at http://www.r-project.org/). Pairwise Student’s t-tests were performed for analysis of statistical significance of differences between two groups. One-way ANOVA (analysis of variance) in combination with post-hoc t-tests were used in order to compare multiple groups. P-values were corrected according to Holm [33]. Statistical significance was accepted for an α-error of less than 5% (p<0.05). P-values are denoted in figures. Statistical signifcance of tumour incidence and testicular atrophy was tested by Fisher’s exact test for which we accepted an α < 0.1. Error bars either denote standard deviation (SD) or standard error of the mean (SEM).

## Results

### Expression of *Nf2* isoforms *in vivo*

**Fig 1:**
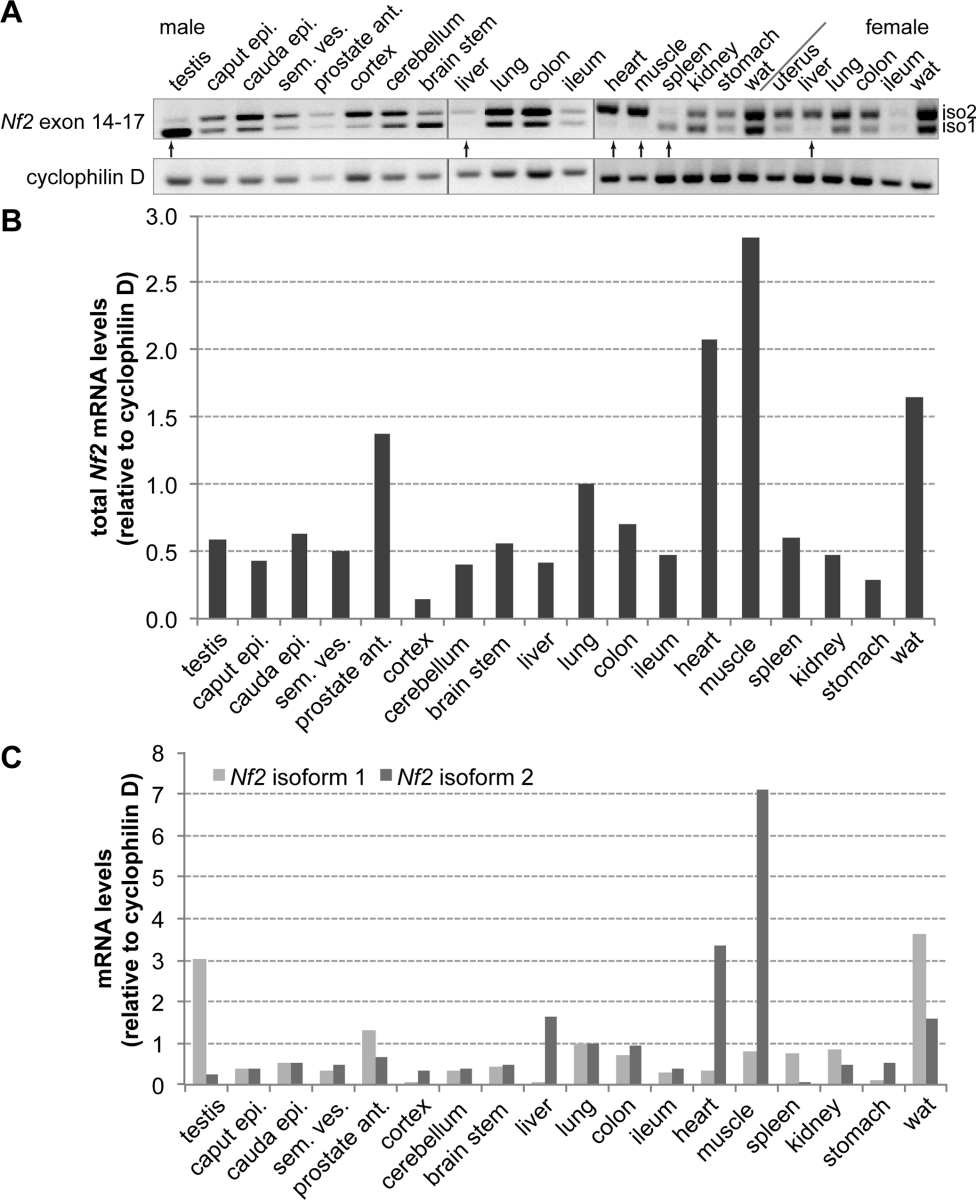
Expression of *Nf2* in major organs of adult mice. **(A)** RT-PCR using primers spanning exon 14 to exon 17 of *Nf2*. Isoform 1 (iso1) mRNA lacks exon 16, making it 45 bp smaller than isoform 2 (iso2). Cyclophilin D levels were used as an mRNA loading control. Arrows denote organs primarily expressing one of the two isoforms (testis, heart, muscle, spleen, liver). **(B)** Determination of mRNA expression levels in male organs using qPCR with primers specific for exon 2 (total *Nf2* mRNA); normalized to lung levels. **(C)** Determination of mRNA expression levels using qPCR with specific primers for isoform 1 mRNA or isoform 2 mRNA; normalized to lung levels. Relative isoform levels were consistent between the two methods (epi., epididymis; sem. ves., seminal vesicles; ant., anterior; wat, white adipose tissue).

Earlier studies provided only limited data regarding *NF2*/*Nf2* isoform expression in humans and rodents [4–7]. In order to investigate the specific *in vivo* function of the two major *Nf2* isoforms and their role during development and organ homeostasis, we first established the specific isoform expression pattern in a wide range of murine organs. This analysis was performed on organs from young adult wildtype C57Bl/6 female and male animals. Expression levels were similar in the two genders. Quantification of *Nf2* isoform mRNA levels by RT-PCR (Fig 1A) revealed *Nf2* expression in all analysed organs. We used the mmNf2ex14-17 primer pair, which spans the alternatively spliced exon 16 and thus amplifies both an isoform 1- and an isoform 2-specific PCR product. In addition, we quantified levels of total *Nf2* expression, as well as isoform 1 and isoform 2 expression by qPCR (Fig 1B and 1C). Prostate, lung, heart, skeletal muscle and white adipose tissue showed a strikingly high expression level of *Nf2* compared to other organs (Fig 1B). We observed a relatively equal split in *Nf2* isoform 1 and isoform 2 expression in most organs; however, some organs showed a predominant expression of either isoform 1 or 2. Specifically, the testis and spleen displayed an isoform 1 preference, while the liver, heart and skeletal muscle preferentially expressed isoform 2 (Fig 1A and 1C).

### Establishment of *Nf2* iso1 ko and iso2 ko animal models

**Fig 2:**
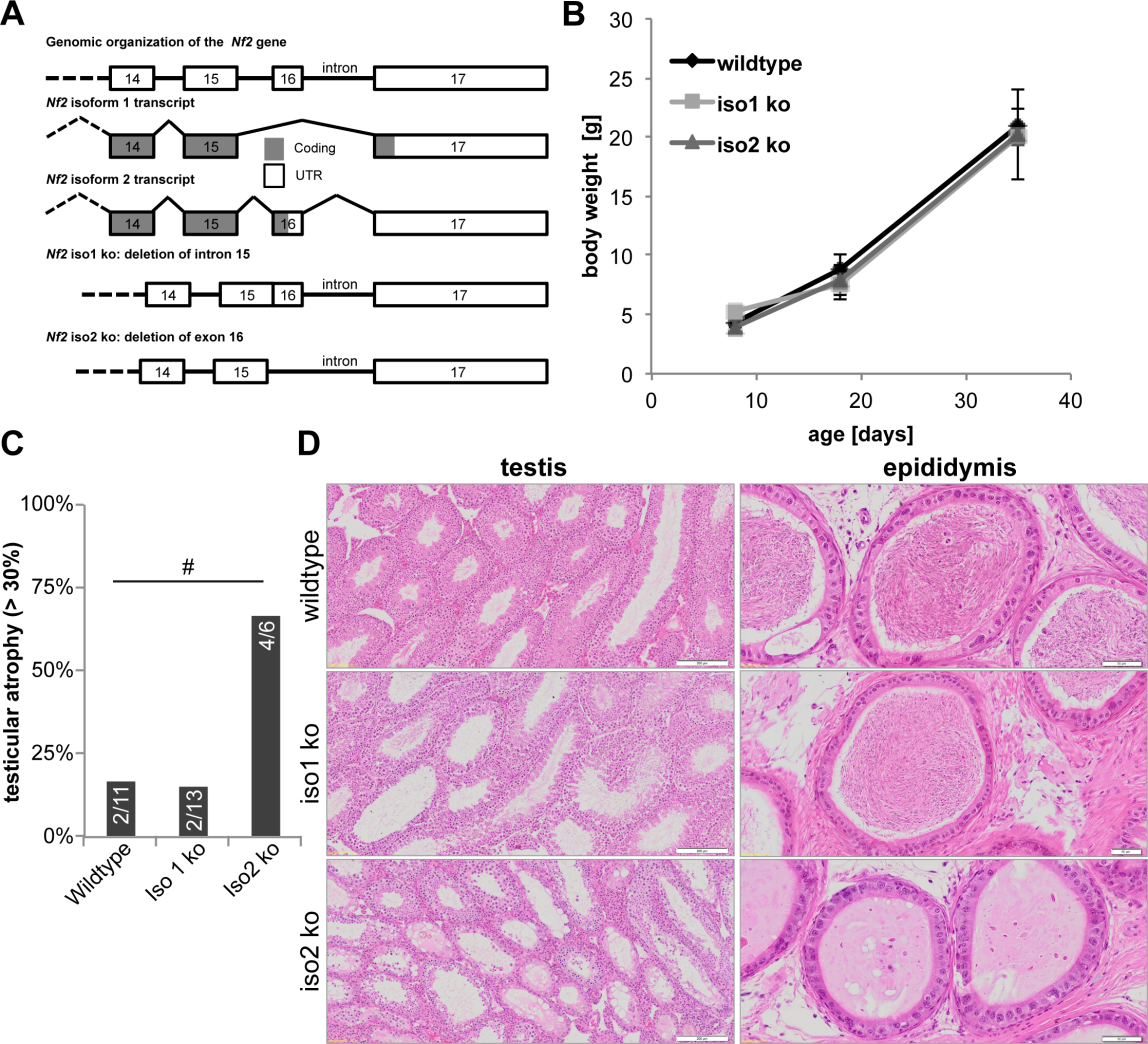
Knockout of *Nf2* isoform 2 causes testicular atrophy in aged mice. **(A)** Organization of the *Nf2* gene locus, splicing of isoform 1 and gene knockout strategy. Note that deletion of one isoform causes expression of the other. **(B)** Body weight of iso1 ko and iso2 ko animals. Development of mice was normal (n = 3 for each genotype and time point). **(C)** Percentage of aged (22–26 months old) animals displaying testicular atrophy in more than 30% of tubules. #: significant on an a α-level of <0.1 (Fisher’s exact test). **(D)** H&E-stained tissue sections showing testicular atrophy and empty epididymis of iso2 ko animals. Scale bar = 200 μm (testis)/ 50 μm (epididymis).

In order to investigate the *in vivo* significance of the two isoforms, we utilised isoform-specific knockout mice generated by M. Niwa-Kawakita in the Giovannini laboratory [17]. In these mice, alternative splicing of *Nf2* exon 16 is altered in such a way that the cells must specifically express one isoform, thus generating a “knockout” (ko) for the other isoform (Fig 2A, S1 Fig). The knockout of isoform 2 is achieved by deletion of exon 16, therefore only isoform 1 can be generated. This mouse line is denoted as iso2 ko. The knockout of isoform 1 is achieved by fusion of exon 15 and exon 16, i.e. deletion of intron 15, which abolishes alternative splicing and thus forces expression of isoform 2. This mouse line is denoted as iso1 ko (Fig 2A).

### Tumour suppressor function of *Nf2* isoform 1 and 2

Gross observation of the iso1 ko and iso2 ko animals did not reveal any obvious phenotype. The animals developed normally without any alterations in growth and body weight gain (Fig 2B). We did not observe increased lethality for the analysed time period (up to 26 months of age) in either iso1 ko or iso2 ko animals of mixed C57Bl/6-FVB and pure C57Bl/6 background. More importantly, we did not detect increased tumour formation in young or old knockout animals as judged from histological stainings of all major organs (Table 4). Therefore, both *Nf2* isoforms must be considered as genuine tumour suppressors, considering heterozygous *Nf2* deletion causes the development of multiple tumours within one year [10]. *In vitro*, both isoforms were equally able to inhibit known Merlin regulated pathways such as the Hippo pathway (S2 Fig) in cell culture assays (also [22–26]). Why then do organs like the liver or the testis predominantely express isoform 2 or isoform 1, respectively? In order to answer this question, we analysed organs expressing primarily one of the two isoforms, i.e. the testis for isoform 1 and liver and skeletal muscle for isoform 2 in the respective isoform knockout animals.

**Table 4:** Tumours observed in aged iso1 ko and iso2 ko animals.

*disease*	*Wildtype (n = 18)*	*Iso1 ko (n = 20)*	*Iso2 ko (n = 11)*
**Fibrosarcoma**	0%	5%	0%
**hepatoma**	11%	5%	0%
**lymphoma**	6%	10%	27%
**kidney polycystic disease**	6%	5%	0%
**lung adenoma**	6%	0%	0%
**kidney invasive adenocarcinoma**	0%	0%	9%
**liver: cirrhosis/chronic hepatitis**	0%	0%	9%

### No muscle or liver phenotype observed in iso2 ko animals

Despite a predominant expression of isoform 2 in skeletal muscle and liver we did not observe any obvious morphological changes of the these organs in iso2 ko animals (S3 Fig). Muscle strength as assessed by the hanging wire test was unaltered in iso2 ko animals (S4 Fig). Knockout of isoform 2 also did not have any effect on Yap and Erk phosphorylation (S4 Fig). However, while Merlin protein levels were unaffected by the isoform switch in the muscle (S4 Fig), the amount of total Merlin increased in the liver of iso2 ko animals compared to wildtype littermates (S4 Fig). In accordance, we noticed an inhibition of Erk phosphorylation (S4 Fig) and a slight increase in liver fat content (S4 Fig). However, despite the observed changes in MAPK signalling, histological analysis did not reveal an altered development or impaired organ homeostasis of the liver (S3 Fig).

### Testicular atrophy in aged *Nf2* iso2 ko mice

First, despite the testis predominantly expressing isoform 1 and only a low level of isoform 2 (Fig 1A and 1C), we surprisingly found a high incidence of intermediate testicular atrophy in aged iso2 ko animals (Fig 2C and 2D). Additonally, the affected animals also displayed a virtual absence of sperm in the epididymis (Fig 2D). We therefore investigated the role of both isoforms in the testis and male reproductive system in more detail.

### Expression of *Nf2* isoform 1 and 2 in the testis and epididymis

**Fig 3:**
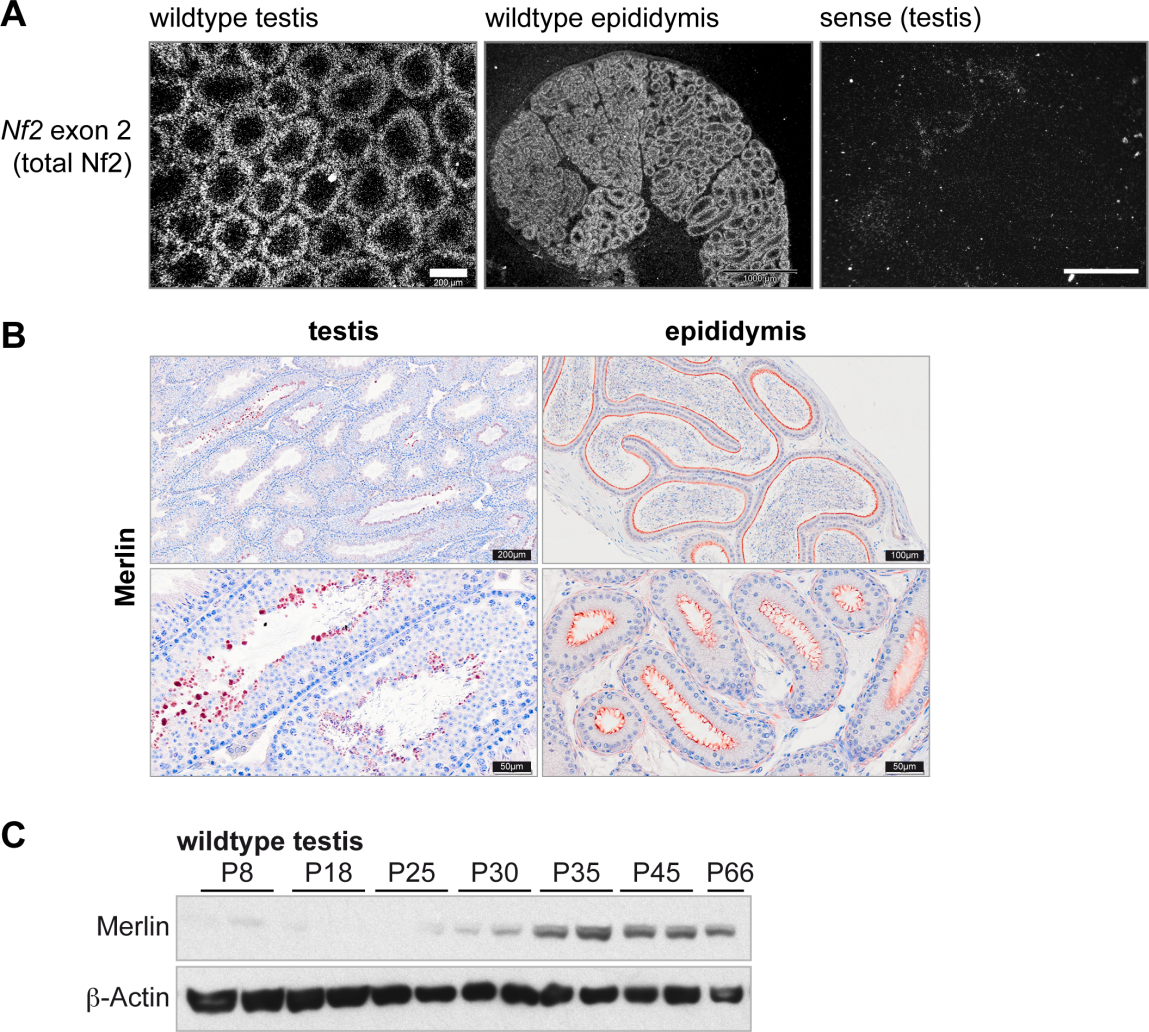
*Nf2* is expressed in the seminiferous and epididymal epithelium. **(A)** *In situ* hybridisation analysis using radioactively labelled probes targeting exons 1–4 of *Nf2*. *Nf2* specific signals were present in the seminiferous tubules but not the interstitium of the testis (scale bar = 200 μm) and in all tubules of the epididymis (scale bar = 1000 μm). **(B)** IHC staining shows Merlin to be abundantly expressed in residual bodies in the testis and at the apical site of the epididymal epithelium. **(C)** Western blot analysis of Merlin protein levels in testicular lysates at different post-natal stages showing elevated expression of Merlin from P30 onwards.

Considering the low expression level of *Nf2* isoform 2 in the testis, we assumed two possibilities: either all cell types express only a small amount of isoform 2 in favour of isoform 1 or a small subset of cell types in the testis express isoform 2. We therefore aimed to identify the cells expressing isoform 1 and 2. We performed *in situ* hybridisation (ISH) analysis to visualize total *Nf2* mRNA distribution pattern in the adult testis, which revealed strong expression of *Nf2* in the seminiferous tubules as indicated by the circle-like staining pattern (Fig 3A). Thus, *Nf2* is expressed in Sertoli cells or germ cells and not in the interstitium, i.e. it is not expressed by Leydig cells. Additionally, we found that *Nf2* is expressed in every tubule of the epididymis (Fig 3A). Immuno histo chemistry (IHC) staining of Merlin using an antibody that recognizes both isoforms confirmed expression in all tubules of the epididymis and revealed an apical localisation of the protein (Fig 3B). The staining pattern in the testis did not mirror the ISH data, however, this suggested an accumulation of Merlin protein in late spermatid stages and most prominently in residual bodies. Analysis of testicular protein levels at different postnatal stages also demonstrated an upregulation of Merlin around P30, at a time when the first elongated spermatids appear (Fig 3C).

**Fig 4:**
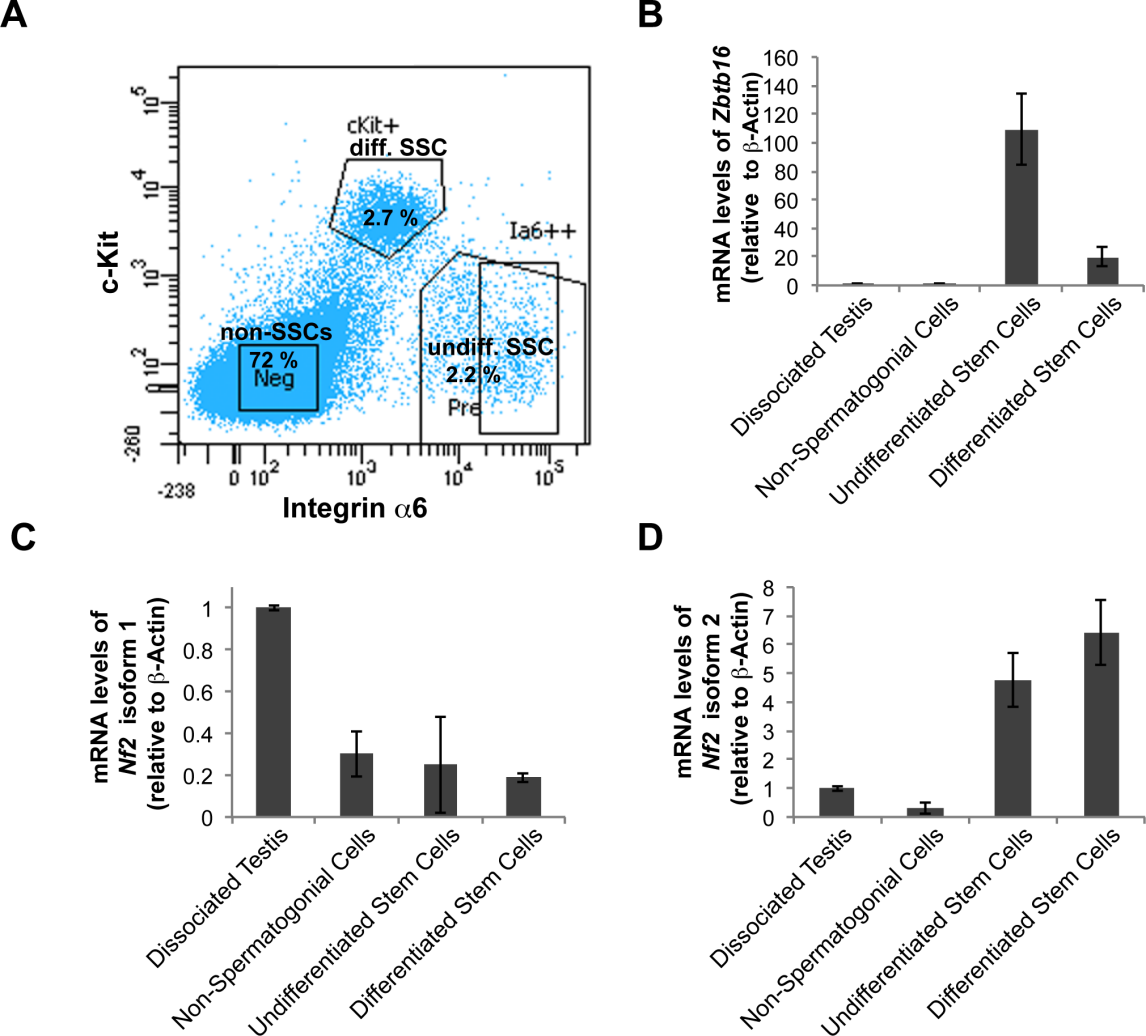
*Nf2* isoform 2 is enriched in spermatogonial stem cells. **(A**) MACS pre-sorted EpCam^+^ cells were FACS sorted according to Integrin α6 and c-Kit expression into non-spermatogonial cells (Integrin α6^–^, c-Kit^–^), undifferentiated spermatogonial stem cells (Integrin α6^+^, c-Kit^–^) and differentiated spermatogonial stem cells (Integrin α6^+^, c-Kit^+^). mRNAs of *Zbtb16*, *Nf2* isoform 1 and *Nf2* isoform 2 were measured in the sorted cell fractions and whole testis lysates by qPCR and normalised to β-actin (*Actb*) levels. **(B)** Stem cell marker *Zbtb16* was highly enriched in the undifferentiated spermatogonia fraction. Analysis of *Nf2* isoform 1 **(C)** and isoform 2 **(D)** mRNA in the sorted cell fractions revealed enrichment of isoform 2 and low isoform 1 expression in spermatogonial stem cells. Error bars in C, D and E denote SDs of technical replications of representative cell isolation.

The exon specific for isoform 2 is only 45 bases long and all exons of isoform 1 are present in isoform 2 mRNA severely limiting ISH probe sensitivity. Therefore, we could not use isoform-specific probes. Instead, we aimed to use the sensitive qPCR technique with specific primers for each isoform. The ISH revealed high *Nf2* expression in the germ cell-Sertoli cell compartment, and since one of the smallest subpopulations of the seminiferous tubules is comprised of spermatogonial stem cells (SSCs), we made use of established SSC enrichment protocols using MACS/FACS [31]. Spermatogonia were MACS pre-sorted for EpCam^+^ cells. Subsequent FACS analysis sorted the cells according to Integrin α6^+^ and c-Kit expression. Cells were sorted into three groups: non-SSCs (Integrin α6^+^, c-Kit^+^), differentiated SSCs (Integrin α6^+^, c-Kit^+^) and undifferentiated SSCs (Integrin α6^+^, c-Kit^+^) (Fig 4A). We used the undifferentiated SSC marker *Zbtb16* to control the purity of the fractions (Fig 4B). Analysis of isoform mRNA expression in the purified cells revealed an interesting pattern of isoform expression in the testis. Spermatogonia (Integrin α6^+^) displayed an almost 20-fold increase in isoform 2 expression compared to non-SSCs (Integrin α6^+^) and a six-fold increase compared to whole testis lysates (Fig 4D). In contrast, isoform 1 expression was highest in the total lysate, thus indicating that this isoform is expressed primarily in a non-SSC compartment, either the Sertoli cells or differentiated germ cells like spermatocytes or spermatids (Fig 4C).

### Sperm analysis of *Nf2* iso1 ko and iso2 ko animals

**Fig 5:**
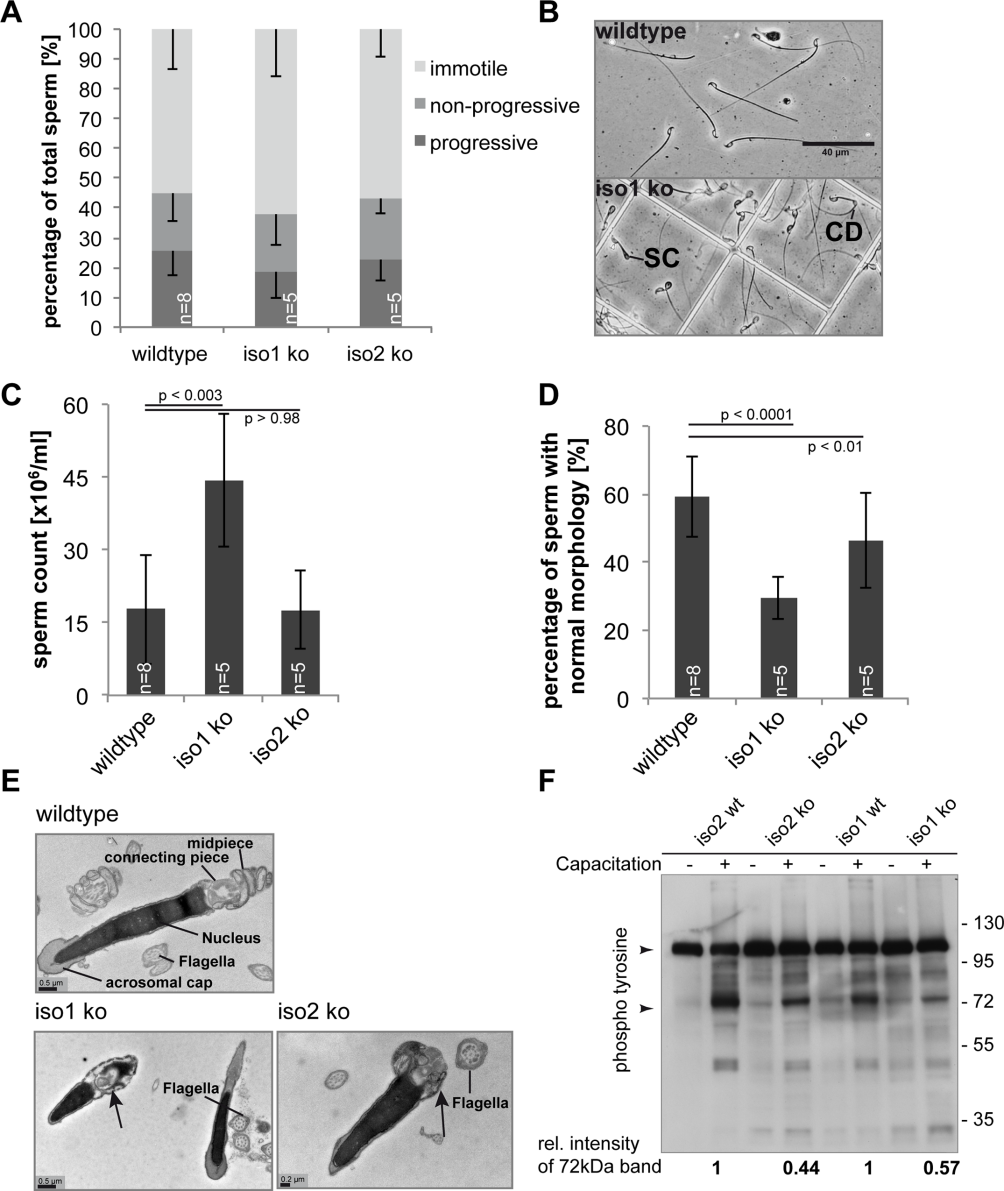
Deletion of either isoform 1 or isoform 2 impairs spermatogenesis. **(A)** Sperm motility was unchanged in both knockouts. **(B)** Demonstration of normal (wildtype) sperm and immature sperm showing sperm coiling (SC) and cytoplasmic droplets (CD) (iso1 ko). Scale bar = 40 μm **(C)** Total count of seminal duct sperm revealed increased sperm numbers in iso1 ko mice. **(D)** Morphological classification of sperm from seminal duct. Both iso1 ko and iso2 ko sperm showed lower sperm quality as measured by cytoplasmic droplets and sperm coiling. **(E)** Electron microscopy of sperm. Iso1 ko and iso2 ko sperm preparations showed normal flagellum morphology but frequently aberrant sperm head morphology as seen by, for example, midpiece deformation (arrows). **(F)** *In vitro* sperm capacitation assay analysed by Western blot phospho tyrosine staining. Both iso1 ko and iso2 ko sperm showed decreased phospho tyrosine levels compared to their littermates (quantification correlated the prominent band around 72 kDa against the constitutively phosphorylated protein band at 110 kDa, arrowheads).

The relative distribution of motile and immotile sperm was similar in the two isoform knockouts (Fig 5A). Surprisingly, the iso1 ko mice produced about twice as much sperm as the wildtype, whereas iso2 ko animals exhibited normal sperm levels (Fig 5C). Iso1 ko and iso2 ko mice displayed reduced sperm quality compared to wildtype animals (Fig 5D), as characterised by sperm coiling (SC) and increased occurrence of cytoplasmic droplets (CD) in vas deferens sperm (Fig 5B). Interestingly, sperm preparations from iso1 ko animals featured an increased percentage of abnormal sperm compared to iso2 ko animals (Fig 5D).

In order to further refine the phenotype of the knockout sperm, we analysed sperm by transmission electron microscopy (EM). While some sperm appeared normal, we detected a wide variety of sperm head defects and abnormal midpiece structures in the atypical sperm of both iso1 ko and iso2 ko animals compared to wildtype littermates (Fig 5E). The flagella seemed to be formed normally and we observed the typical “9+2” formation of microtubules in flagellum cross-sections [34].

### Capacitation reaction of sperm deficient in *Nf2* isoform 1 or 2

Finally, we aimed to investigate whether the defects found in the knockout sperm of both isoform animals would have any consequences on sperm function. After ejaculation, sperm undergo capacitation, enabling the acrosome reaction and hypermotility, which are mandatory for successful fertilisation [35]. We tested the sperm in an *in vitro* capacitation assay. Stimulation of wildtype sperm with BSA and NaHCO_3_ leads to activation of the capacitation reaction, visible by an increase in tyrosine phosphorylated proteins in Western blot analysis. We found that sperm from both isoform knockout mice displayed the typical tyrosine phosphorylation pattern seen in wildtypes, but with a reduction in intensity best seen in the prominent bands above 70 kDa, which were reduced to approximately 50% of that of wildtype littermates (Fig 5F). Thus, the knockout of either Merlin isoform has a detrimental effect on sperm function.

### Fertility impairment varied with genetic background

**Fig 6:**
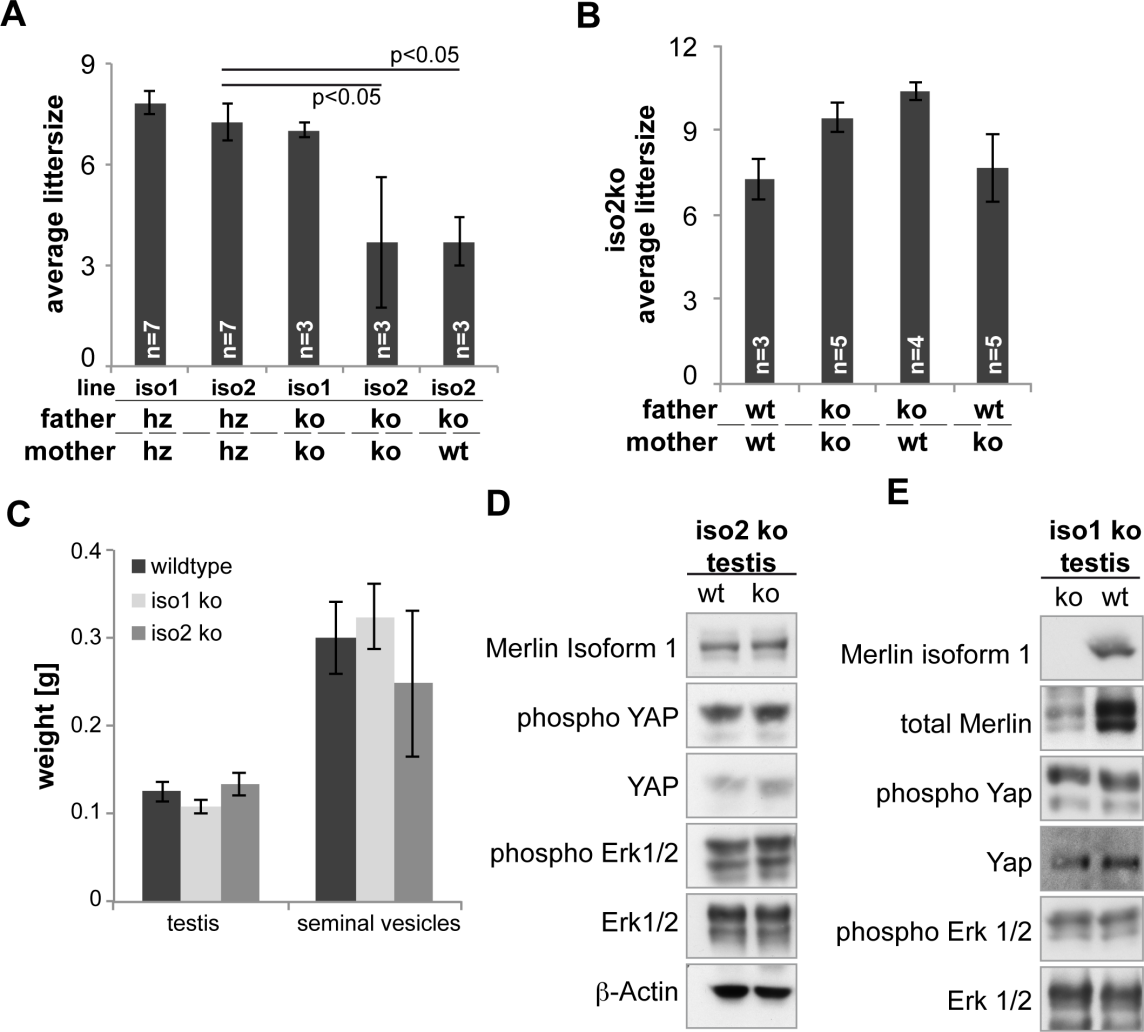
Classical merlin regulated pathways are unaltered in ko testes. Average littersize of different breeding combinations over a period of 6 months, starting at 2 months of age, on a C57Bl/6 **(A)** and a mixed C57Bl/6-FVB/NJ **(B)** genetic background revealed a background dependent impact of the isoform 2 knockout on fertility. **(C)** Testis and seminal vesicle weight of both knockout lines was unchanged at 3 months (n = 5). **(D)** and **(E)** Western blot analysis of whole testis lysates indicated unaltered Hippo (phospho Yap) and MAPK (phospho Erk) signalling in both knockouts. Knockout of isoform 1 led to altered merlin protein levels in iso1 ko testis **(E)**.

Decreased sperm functionality should also lead to an impairment of fertility. We investigated fertility of the knockout lines by recording the littersize over a timespan of 6 months for different combinations of breeding pairs. Despite lower sperm quality in both iso1 ko and iso2 ko animals, we observed a reduction in litter size only in iso2 ko breedings, which could be attributed to the males (Fig 6A). However, fertility was only impaired in animals on a C57Bl/6 background, animals on a mixed C57Bl/6-FVB/NJ background did not show any signs of decreased fertility (Fig 6B).

In sum, deletion of *Nf2* isoform 1 or 2 led to sperm defects and in the case of iso2 ko animals to impaired fertility. Therefore, we next investigated whether this phenotype orignated in the testis or the epididymis.

### Morphology of testis and epididymis

We first analysed testis weight as an indicator of functional spermatogenesis. Additionally, since the isoform knockout is total and applies to all cells, the hypothalamus-pituitary-testis (HPT) axis might be affected by the knockout and, via disturbed hormone levels, influence spermatogenesis. As a read-out for intact HPT homeostasis, we used seminal vesicle weight which correlates tightly with active testosterone levels in the mouse [36]. The testes’ and seminal vesicles’ weight of both iso1 ko and iso2 ko animals was unchanged in comparison to wildtypes at 3 months of age (Fig 6C), indicating functional spermatogenesis and normal testosterone levels.

**Fig 7:**
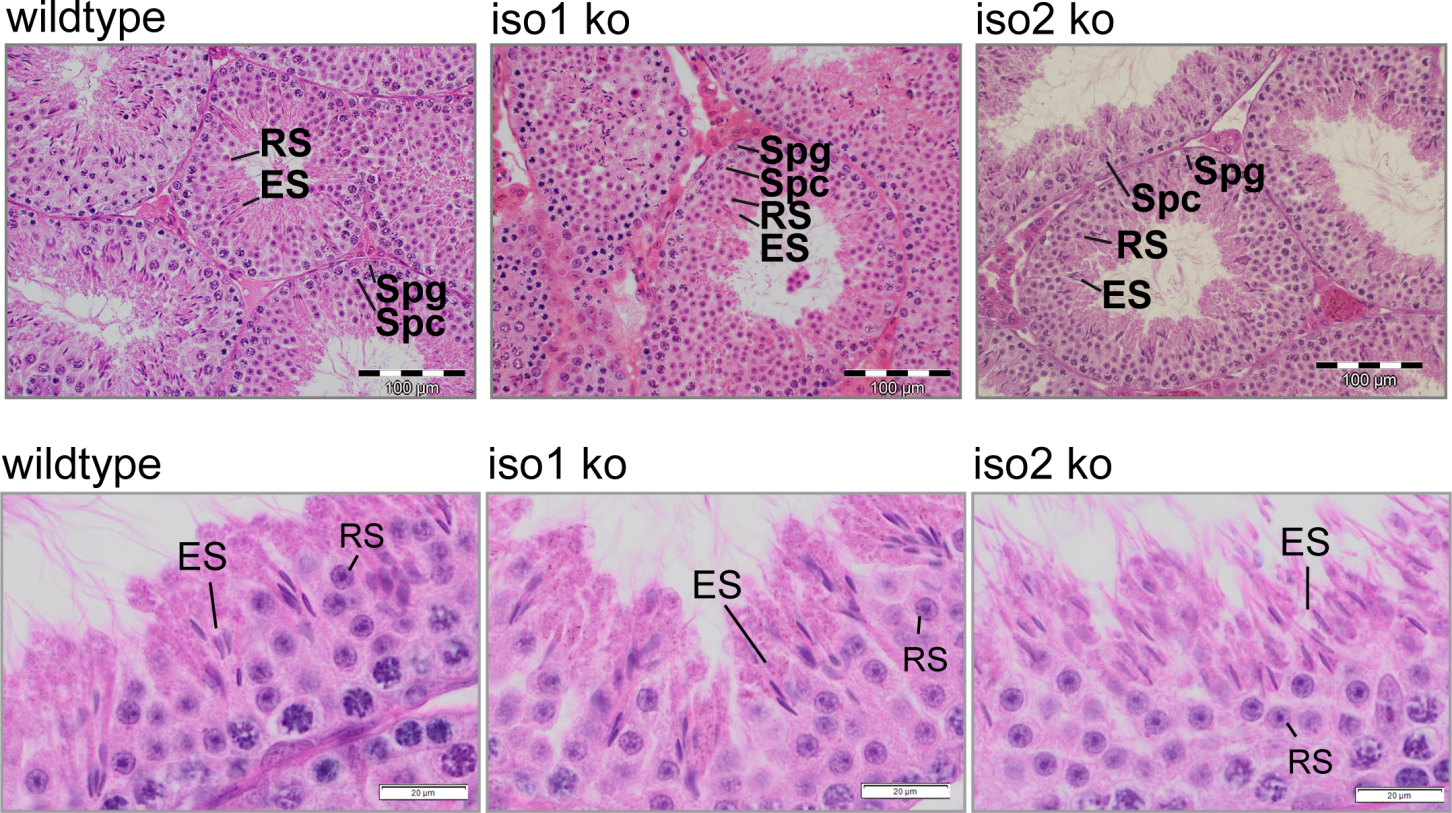
Unchanged histology in knockout testes. H&E stained testes from 3 months old iso1 ko and iso2 ko mice show normal sperm head morphology of stage 15 spermatids. (RS, round spermatids; ES, elongated spermatids). Scale bar = 20μm.

Since testis weight was unchanged sperm production in the testis was judged to be grossly normal. We therefore investigated the testicular histology of 3 months old iso1ko and iso2ko animals (Fig 7). However, we found no pathological alterations nor other hints for the cause of the sperm defects in young adults, indicating that the development of atrophy in iso2 ko testes is strictly age dependent and likely independent of the sperm defect.

Additonally, we analysed the status of the two most prominent Merlin-regulated pathways, the MAPK and Hippo pathways. However, no alterations of Erk phosphorylation, as a measurement of MAPK activity, or Yap phosphorylation, as an indicator of Hippo signalling, were observed (Fig 6D and 6E). Interestingly, while iso2 ko testes showed similar Merlin isoform 1 levels as the wildtype (Fig 6D), deletion of isoform 1 caused a decrease in total Merlin levels (Fig 6E).

### Increased lysosome abundance in the caput epididymis

**Fig 8:**
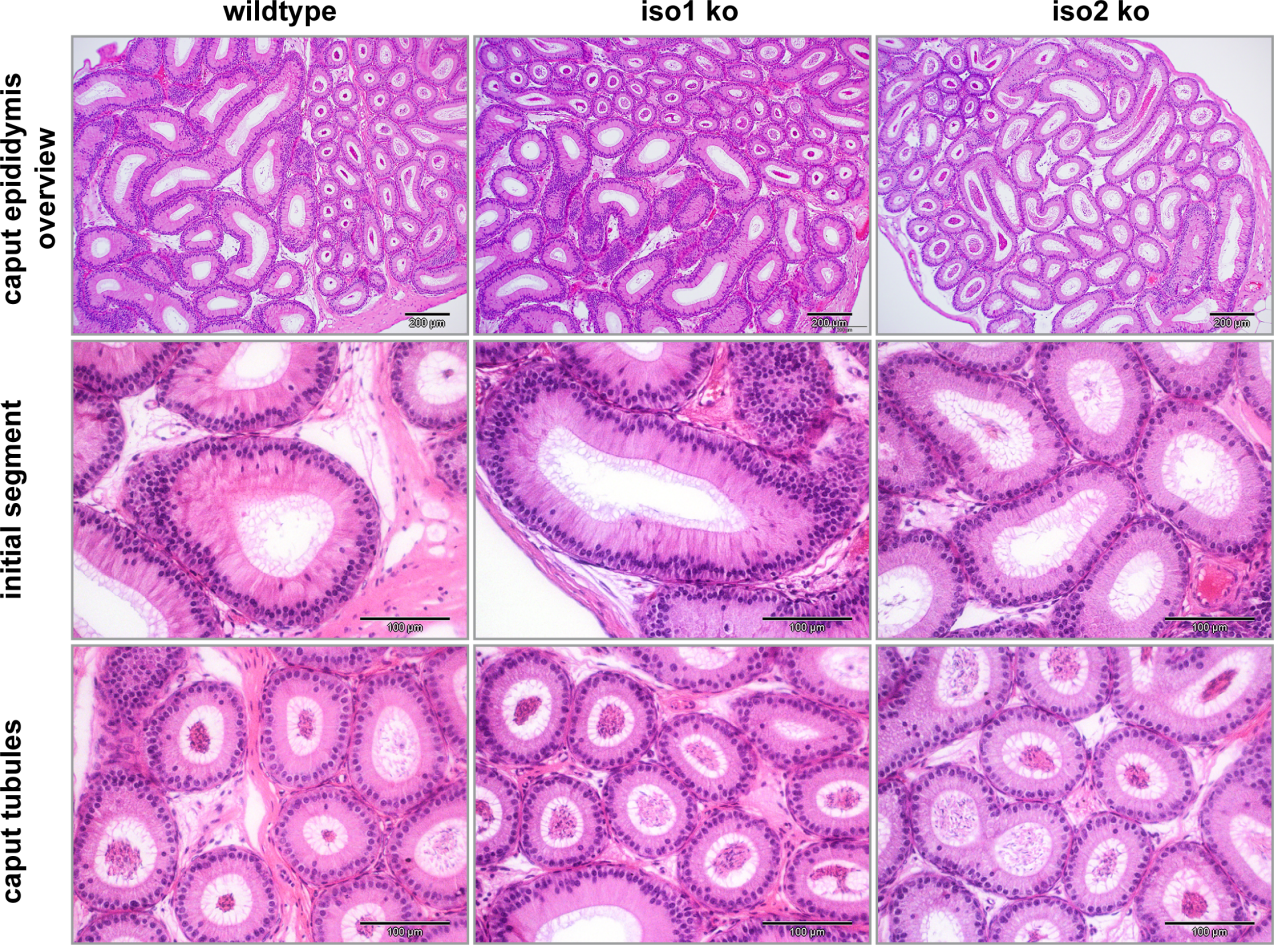
Epididymal vesicle formation is altered in ko animals. H&E stained sections showed normal histology of iso1 ko and iso2 ko epidiymises. (scale bars = 200 μm (overview)/ 100μm (lower panels)).

**Fig 9:**
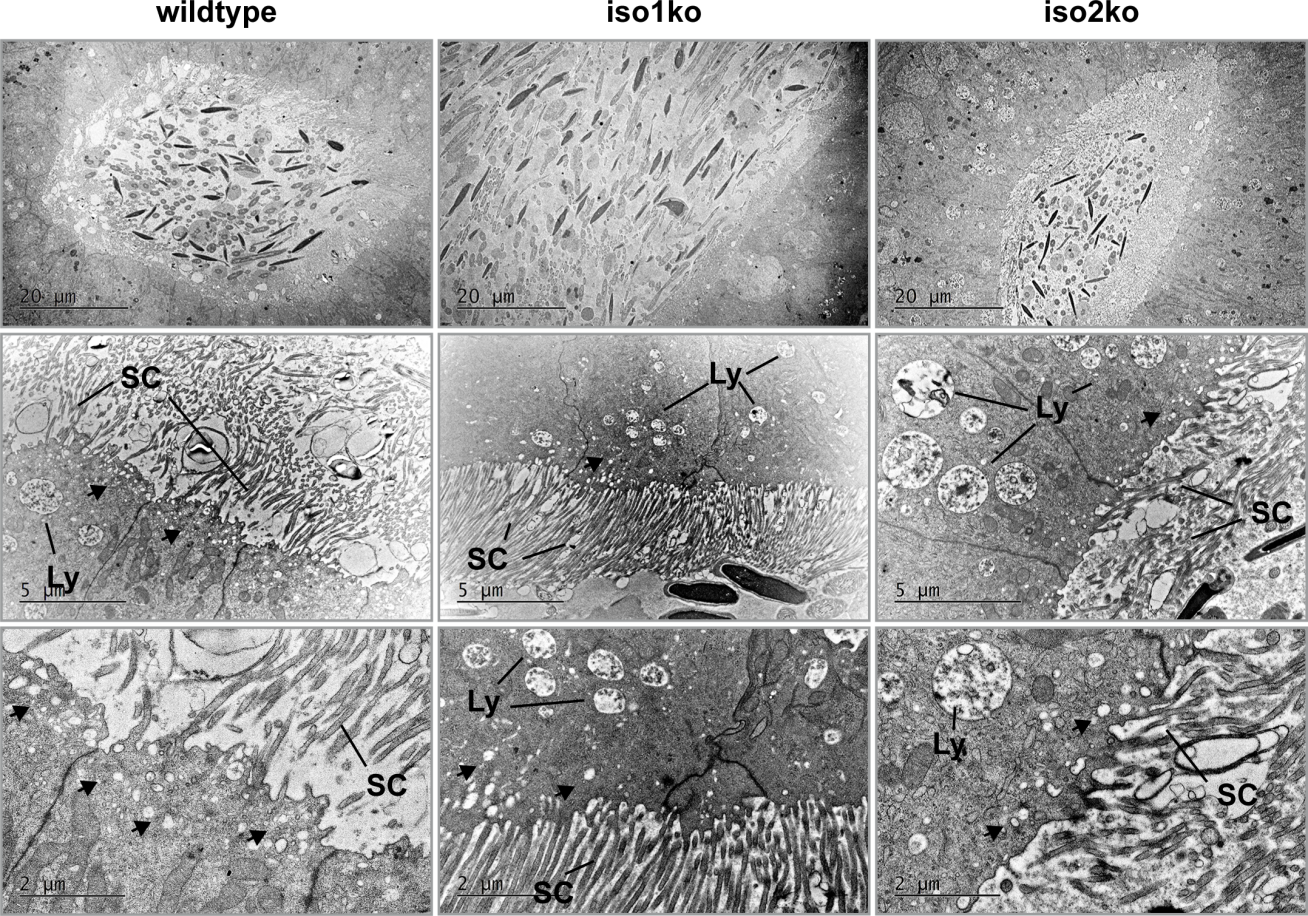
Increased lysosome formation in epididymis of ko animals. Electron micrographs of the caput epididymis revealed increased lysosome (Ly) formation but a lower abundance of small secretory vesicles (arrows). Stereocilia (SC) appeared normal in morphology and numbers.

We found no evidence for altered spermatogenesis in the testis despite a clear defect in sperm maturation and an increased sperm count of iso1 ko animals. However, both Merlin isoforms were expressed in the epididymis (Fig 1), which plays a vital role in the maturation of sperm. Additionally, sperm maturation is regulated by the microenvironment of the epididymal fluid [37, 38]. Microvilli of epithelial cells, the stererocilia, harbor various proton pumps and ion channels that allow for modification of fluid parameters [39]. In general, microvilli structure and maintenance requires the proteins Ezrin, Paxillin and filamentous Actin [40, 41]. Because Merlin belongs to the Ezrin, Radixin, Moesin (ERM) familiy of actin-linker proteins and is known to bind Paxillin we considered that loss of Merlin isoforms might alter the microvilli structure and function. Therefore, we investigated the epididymis as a potential origin of the sperm defects. Although histological analysis of the epididymis did not reveal morphological changes (Fig 8), electron microscopy uncovered increased lysosome formation and a lower abundance of small secreted vesicles in the caput epididymis (Fig 9). On the other hand, microvilli structure and abundance was judged to be normal.

In summary, we observed testicular atrophy in old iso2 ko animals and sperm defects in young animals of both knockout lines. While we found testicular appeareance to be normal in 3 months old animals, we discovered evidence for alterations of vesicle and lysosome abundance in the epididymal epithelium. It is possible that this has an effect on sperm maturation during the epididymal transit.

## Discussion

Various mouse models with conditional or total deletion of *Nf2* have provided ample evidence for an indispensable role of Merlin in tissue homeostasis regulation and development [9–11, 13–15, 42]. Although *Nf2* is widely expressed during mammalian embryogenesis [3], its role during development is still poorly understood. In addition, the *in vivo* role of alternative splicing of the *Nf2* gene into two major Merlin isoforms is currently unknown. In this study, we examined the functional uniqueness of the *Nf2* isoforms *in vivo* by employing mouse models genetically altered to express only one isoform. We provide for the first time *in vivo* evidence for equal tumour suppressor function of isoform 1 and isoform 2. Neither isoform knockout affected animal development or caused increased tumour formation in the mice in our hands, both on a mixed FVB-Bl6 as well as a pure C57Bl/6 background. Additionally, we add spermatogenesis to the list of cellular processes critically dependent on the fine-tuned regulation of *Nf2*/Merlin expression. Surprisingly, deletion of either isoform impaired sperm quality and caused decreased sperm functionality.

### Equal tumour suppressor activities of *Nf2* isoform 1 and isoform 2

We demonstrate in this work that both Merlin isoform 1 and isoform 2 are fully functional tumour suppressors. Our finding that several organs specifically expressed predominantely one isoform in adult mice (Fig 1) supports the hypothesis of equal tumour suppressor potentials of both isoforms, since these organs have to rely on only one isoform to control growth signalling. It is important to note that these organs do not simply use other tumour suppressors for growth regulation. The liver mainly expresses *Nf2* isoform 2 without any liver tumours observed, even in old iso2 ko mice. However, deletion of *Nf2* in the liver reproducibly leads to a massive increase in liver mass and eventual tumours within one year [11, 13]. Additionally, we did not observe increased tumour formation in any organ of iso1 ko or iso2 ko animals up to 26 months of age, regardless of its initial *Nf2* isoform expression pattern, although heterozygous inactivation of *Nf2* in mice causes the development of multiple tumours within one year [10].

Surprisingly, our results so far indicate that both isoforms can functionally compensate the loss of the other almost entirely, even in organs originally expressing primarily a single isoform such as the liver and skeletal muscle, the exception being the testes. It is possible, however, that injury models of liver or muscle would retrieve a regeneration response phenotype in the isoform knockout mouse models similar to deletion of *Nf2* in adult liver [11]. Additionally, since even NF2 disease tumours are thought to arise only after multiple mutation hits [43], an alternate model would be the simultaneous deletion of other tumour suppressors to lower the tumour barrier and thus elicit differential tumour formation behaviour in iso1ko and iso2ko mice. A potential candidate for this approach would be the *Cdkn2a* gene (encoding P16^Ink4a^ and P19^ARF^) that has recently been shown to genetically interact with *Nf2* mutations in the development of mesothelioma [44].

### Inactivation of either *Nf2* isoform impairs spermatogenesis

Unexpectantly, we found that deletion of either isoform of the mouse *Nf2* gene disturbed the formation of functional sperm (Fig 5). While the deletion of isoform 1 caused a more severe decrease in functional sperm, deletion of isoform 2 led to testicular atrophy in aged animals. It is possible that germ cell loss is iso2 ko mice is caused by aberrant spermatogonial stem cell maintenance as *Nf2* isoform 2 was enriched in the testicular stem cell population. Interestingly, we observed impaired fertility only for iso2 ko animals and only on a C57Bl/6 background, not on a mixed C57Bl/6-FVB/NJ background. Noteworthy though, it has previously been reported that inactivation of protein 4.1G, which belongs to the protein 4.1 superfamily together with Merlin, displays impaired male fertility only on a mixed 129SV-C57Bl/6 background but not on a pure C57Bl/6 background [45]. The authors of this study suggest “*the presence of major modifier genes that influence 4*.*1G function*” on different backgrounds. Furthermore, Merlin has already been described to play a role in *Drosophila melanogaster* spermatogenesis in regulating mitochondria morphogenesis of maturing sperm [46, 47]. Additionally, Giovannini et al. reported that accidental deletion of *Nf2* in the testis causes infertility in mice [42]. Collectively, the existent data strongly argue for a vital role of Merlin during spermatogenesis.

The *Nf2* isoforms are expressed at different stages of spermatogenesis, namely isoform 2 in spermatogonia and isoform 1 in differentiated germ cells and possibly Sertoli cells (Fig 4). Our data additionally suggests that Merlin protein levels peak during the final phases of spermatid elongation but that Merlin is then concentrated in residual bodies, as sperm in the epididymis displayed only weak Merlin immuno-reactivity. It is therefore likely that the singular action of each isoform plays a vital role in spermatogenesis regulation. Importantly, comparison of seminal vesicles’ weight as an indicator of testosterone levels excluded disturbance of hormonal regulation due to the knockout of the isoforms in all cells as a cause of spermatogenesis impairment. Rossi et al. reported in 2004 that *Nf2* is three-fold enriched in mouse spermatocytes compared to spermatogonia [48]. This supports our finding that isoform 1, the predominant testicular *Nf2* isoform, is expressed in later germ cell stages. It is unclear, however, whether Sertoli cells also express *Nf2*, since our FACS and ISH data do not distinguish between Sertoli cells and later germ cell stages. In conclusion, these results clearly demonstrate that fine-tuned expression and regulation of *Nf2*/Merlin are important for spermatogenesis in the testis. We could not, however, satisfactorly conclude the molecular cause of the sperm defect. It would therefore be interesting to determine whether conditional deletion of *Nf2* in Sertoli cells or germ cells can recapitulate the sperm phenotype of the isoform deletion mouse models, potentially providing better tools to study the molecular role of Merlin in the testis.

While visible sperm head aberrations (Fig 5E) are usually due to sperm differentiation defects in the testis, sperm concentration changes and increased sperm coiling can be caused by alterations of the epididymis, specifically its secretory behaviour controlling osmolarity and volume of the epididymal fluids [37, 38]. Regulation of fluid parameters depends on pumps and channels present in microvilli[39]. Microvilli are structured by Actin filaments which are stabilized by Paxillin and the ERM proteins [40, 41], all of which have been shown to interact with Merlin [49–51]. Furthermore, the microvilli actin structure is anchored to the terminal web, which among others, is composed of spectrin proteins. β2-Spectrin has been shown to preferentially interact with Merlin isoform 2 [52], hence alterations of *Nf2* isoform expression may impact epididymal microvilli, fluid absorption and sperm transport. Our electron microscopy data do not support alterations of the microvilli structure of epididymal epithelial cells, however we noticed an increase in lysosomes and a decrease in small secretory vesicles. While we have no data on epididymal fluid parameters, one can speculate that loss of *Nf2* isoforms impairs epithelial secretion and absorption processes, which in turn could promote sperm coiling and might affect sperm concentration parameters as seen for the iso1 ko animals (Fig 5). On the other hand, the above mentioned proteins are also part of the apical ectoplasmic specialisation [53, 54]. Disturbance thereof could likewise cause sperm differentiation defects and could potentially explain the observed testicular atrophy in iso2 ko animals at older age (Fig 2D). Furthermore, increased sperm production does not necessarily lead to a proportional increase in testis weight [55]. Increased sperm concentration observed in iso1 ko mice might therefore also be explained by pathological testicular spermatogenesis. Finally, altered epididymal fluid transport could be the cause for the observed testicular atrophy in aged iso2 ko mice as occlusion of e.g. the efferent ducts is known to lead to long-term tubular atrophy [56].

It is unclear whether the complete inactivation of one isoform is comparable to the heterozygous deletion of *NF2*, as observed in NF2 patients. Complete loss of one isoform might be more obstructive to spermatogenesis than a gene dosage decrease of both isoforms. Moreover, we noticed a change in total Merlin levels both after knockout of isoform 2 in the liver and isoform 1 in the testis, further shrouding the effect of single isoform action. Thus, we cannot presently conclude that spermatogenesis is likely to be affected by inheritance of *NF2* mutations in humans.

## Conclusion

The functional role of Merlin on the molecular level appears to be complex. Various studies reported that Merlin plays a role in the formation of adherens junctions, the distribution of cell-surface receptors, and regulation of a diverse array of signalling cascades such as the MAPK-, Rac-PAK, mTORC1 and Hippo-YAP pathways (reviewed by [28]). In addition, *Nf2* encoding for two different Merlin isoforms increases the potential interactions even further. We found no evidence for isoform-specific regulation of Hippo and MAPK signalling in iso1 ko or iso2 ko tissues, with the exception of the liver. However, we attribute the increase in Erk phosphorylation to the general decrease in Merlin abundance in the iso2 ko liver.

While the signalling pathways responsible for Merlin’s complex tumour suppressor function remain controversial, our understanding of Merlin as a general tumour suppressor and regulator of organogenesis is becoming more apparent. In summary, we present new *in vivo* evidence for equal tumour suppressor qualities of Merlin isoform 1 and isoform 2. Additionally, isoform substitution does not lead to major developmental disorders. One exception is spermatogenesis, which is sensitive to *Nf2* gene disruption independent of the targeted isoform, thus revealing a novel regulatory role of Merlin in the male reproductive system.

## Supporting Information

S1 Fig Analysis of *Nf2* mRNA levels in iso1 ko and iso2 ko organs. **(A)** qPCR analysis of *Nf2* expression in testes demonstrating the specificity of the isoform primers used. Deletion of isoform 1 clearly increased isoform 2 mRNA levels in the testis (n = 2 for each genotype). **(B)** qPCR analysis of iso2 ko liver *Nf2* mRNA showed successful deletion of isoform 2 mRNA, but in contrast to the clear induction of isoform 1 protein levels, mRNA levels remained similar to the wildtype (n = 3 for each genotype). Note that isoform 1 and 2 do not add up to total *Nf2* levels due to the use of a dT_18_ primer, lessening the amount of cDNA containing the 5’ mRNA region. In all cases the total level of *Nf2* mRNA was reduced compared to the wildtype. However, Merlin protein levels did not correspond clearly to the mRNA levels (Fig 6D and 6E, S3 Fig).
(TIF)

S2 Fig Merlin isoform 1 and isoform 2 are equally able to inhibit Yap. **(A)** Expression of isoform 1 or 2 decreased Yap stability in the human hepatoma cell line C3A. Western blot analysis using isoform specific antibodies generously supplied by D. Gutmann, described in Schulz et al. 2013. **(B)** Expression of either isoform suppressed activity of the YAP-driven CTGF promoter in *Nf2*-/- SC4 schwannoma cells indicating Hippo pathway activation by both merlin isoforms. Firefly luciferase reporter assays normalized to TK-promoter driven luciferase activity. (0: non-transfected control, VC: vector control, FL: full length protein, c-term: carboxy terminal half of Merlin.
(TIF)

S3 Fig Histology of iso2 ko liver and skeletal muscle. H&E stained histological sections of skeletal muscle **(A)** and liver **(B)** of adult (3–6 months) old animals. No pathological changes were observed. (scale bars = 100 μm)
(TIF)

S4 Fig Male iso2 ko liver shows increased fat content. **(A)** Muscle strength as assessed in a hanging wire test was not affected by either knockout (n = 4 for each genotype). **(B)** Liver fat content was visualized by Oil Red O staining and categorized independently by AZ and SM. The median liver fat content of male iso ko mice was slightly increased (Mann-Whitney U-test, #: significance level α<0.10, n(wt) = 8, n(iso2 ko) = 9). **(C)** Western blot analysis of iso2 ko muscle showed similar Hippo (phospho Yap) and MAPK (phospho Erk) activity in knockout and wildtype. **(D)** Loss of isoform 2 in liver decreased Erk phosphorylation in Western blot analysis. Western blot analysis of muscle tissue **(E)** confirmed similar total merlin levels in both knockouts, whereas the level of total Merlin was upregulated in iso2 ko liver **(F)**.
(TIF)

## References

[1] Asthagiri AR, Parry DM, Butman JA, Kim HJ, Tsilou ET, Zhuang Z, et al Neurofibromatosis type 2. Lancet. 2009;373(9679):1974–86. Epub 2009/05/30. 10.1016/S0140-6736(09)60259-2 S0140-6736(09)60259-2 [pii]. . 19476995 PMC4748851

[2] Ruttledge MH, Rouleau GA. Role of the neurofibromatosis type 2 gene in the development of tumors of the nervous system. Neurosurg Focus. 2005;19(5):E6 16398470 10.3171/foc.2005.19.5.7

[3] Akhmametyeva EM, Mihaylova MM, Luo H, Kharzai S, Welling DB, Chang LS. Regulation of the neurofibromatosis 2 gene promoter expression during embryonic development. Developmental dynamics: an official publication of the American Association of Anatomists. 2006;235(10):2771–85. 10.1002/dvdy.20883 . 16894610

[4] Gronholm M, Teesalu T, Tyynela J, Piltti K, Bohling T, Wartiovaara K, et al Characterization of the NF2 protein merlin and the ERM protein ezrin in human, rat, and mouse central nervous system. Molecular and Cellular Neuroscience. 2005;28(4):683–93. 10.1016/j.mcn.2004.11.014 . 15797715

[5] Gutmann DH, Wright DE, Geist RT, Snider WD. Expression of the neurofibromatosis 2 (NF2) gene isoforms during rat embryonic development. Human Molecular Genetics. 1995;4(3):471–8. Epub 1995/03/01. . 7795605 10.1093/hmg/4.3.471

[6] Bianchi AB, Hara T, Ramesh V, Gao J, Klein-Szanto AJ, Morin F, et al Mutations in transcript isoforms of the neurofibromatosis 2 gene in multiple human tumour types. Nature Genetics. 1994;6(2):185–92. Epub 1994/02/01. 10.1038/ng0294-185 . 8162073

[7] Huynh DP, Nechiporuk T, Pulst SM. Alternative transcripts in the mouse neurofibromatosis type 2 (NF2) gene are conserved and code for schwannomins with distinct C-terminal domains. Human Molecular Genetics. 1994;3(7):1075–9. Epub 1994/07/01. . 7981675 10.1093/hmg/3.7.1075

[8] Dunbar Schroeder R, Angelo LS, Kurzrock R. NF2-merlin in hereditary neurofibromatosis 2 versus cancer—biologic mechanisms and clinical associations. Oncotarget. 2013:1–11. 24393766 10.18632/oncotarget.1557 PMC3960189

[9] McClatchey AI, Saotome I, Ramesh V, Gusella JF, Jacks T. The Nf2 tumor suppressor gene product is essential for extraembryonic development immediately prior to gastrulation. Genes & Development. 1997;11(10):1253–65. 10.1101/gad.11.10.1253 9171370

[10] McClatchey AI, Saotome I, Mercer K, Crowley D, Gusella JF, Bronson RT, et al mice heterozygous for a mutationo at the nf2 tumor suppressor locus develop a range of highly metastatic tumors. Genes & Development. 1998;12:1121–33. 9553042 10.1101/gad.12.8.1121 PMC316711

[11] Benhamouche S, Curto M, Saotome I, Gladden AB, Liu CH, Giovannini M, et al Nf2/Merlin controls progenitor homeostasis and tumorigenesis in the liver. Genes & Development. 2010;24(16):1718–30. 10.1101/gad.1938710 20675406 PMC2922501

[12] Yi C, Kissil JL. Merlin in organ size control and tumorigenesis: Hippo versus EGFR? Genes & Development. 2010;24(16):1673–9. 10.1101/gad.1964810 20713513 PMC2922497

[13] Zhang N, Bai H, David KK, Dong J, Zheng Y, Cai J, et al The Merlin/NF2 tumor suppressor functions through the YAP oncoprotein to regulate tissue homeostasis in mammals. Developmental cell. 2010;19(1):27–38. 10.1016/j.devcel.2010.06.015 20643348 PMC2925178

[14] Gladden AB, Hebert AM, Schneeberger EE, McClatchey AI. The NF2 tumor suppressor, Merlin, regulates epidermal development through the establishment of a junctional polarity complex. Developmental cell. 2010;19(5):727–39. 10.1016/j.devcel.2010.10.008 21074722 PMC3033574

[15] Larsson J, Ohishi M, Garrison B, Aspling M, Janzen V, Adams GB, et al Nf2/merlin regulates hematopoietic stem cell behavior by altering microenvironmental architecture. Cell Stem Cell. 2008;3(2):221–7. 10.1016/j.stem.2008.06.005 . 18682243 PMC4197168

[16] Schulz A, Geissler KJ, Kumar S, Leichsenring G, Morrison H, Baader SL. Merlin inhibits neurite outgrowth in the CNS. Journal of Neuroscience. 2010;30(30):10177–86. Epub 2010/07/30. 10.1523/JNEUROSCI.0840-10.2010 30/30/10177 [pii]. . 20668201 PMC6633373

[17] Schulz A, Baader SL, Niwa-Kawakita M, Jung MJ, Bauer R, Garcia C, et al Merlin isoform 2 in neurofibromatosis type 2-associated polyneuropathy. Nature Neuroscience. 2013 Epub 2013/03/05. 10.1038/nn.3348 nn.3348 [pii]. . 23455610

[18] Golovnina K, Blinov A, Akhmametyeva EM, Omelyanchuk LV, Chang LS. Evolution and origin of merlin, the product of the Neurofibromatosis type 2 (NF2) tumor-suppressor gene. BMC Evolutionary Biology. 2005;5:69 Epub 2005/12/06. 1471-2148-5-69 [pii] 10.1186/1471-2148-5-69 16324214 PMC1315344

[19] Gutmann DH, Sherman L, Seftor L, Haipek C, Hoang Lu K, Hendrix M. Increased expression of the NF2 tumor suppressor gene product, merlin, impairs cell motility, adhesionand spreading. Human Molecular Genetics. 1999;8(2):267–75. Epub 1999/02/05. ddc028 [pii]. . 9931334 10.1093/hmg/8.2.267

[20] Sherman L, Xu HM, Geist RT, Saporito-Irwin S, Howells N, Ponta H, et al Interdomain binding mediates tumor growth suppression by the NF2 gene product. Oncogene. 1997;15(20):2505–9. Epub 1997/12/12. 10.1038/sj.onc.1201418 . 9395247

[21] Gavilan HS, Kulikauskas RM, Gutmann DH, Fehon RG. In Vivo Functional Analysis of the Human NF2 Tumor Suppressor Gene in Drosophila. PloS one. 2014;9(3):e90853 Epub 2014/03/07. 10.1371/journal.pone.0090853 24595234 PMC3942481

[22] Yi C, Troutman S, Fera D, Stemmer-Rachamimov A, Avila JL, Christian N, et al A tight junction-associated Merlin-angiomotin complex mediates Merlin's regulation of mitogenic signaling and tumor suppressive functions. Cancer cell. 2011;19(4):527–40. 10.1016/j.ccr.2011.02.017 21481793 PMC3075552

[23] James MF, Han S, Polizzano C, Plotkin SR, Manning BD, Stemmer-Rachamimov AO, et al NF2/merlin is a novel negative regulator of mTOR complex 1, and activation of mTORC1 is associated with meningioma and schwannoma growth. Molecular and Cellular Biology. 2009;29(15):4250–61. 10.1128/MCB.01581-08 19451225 PMC2715803

[24] Laulajainen M, Muranen T, Carpen O, Gronholm M. Protein kinase A-mediated phosphorylation of the NF2 tumor suppressor protein merlin at serine 10 affects the actin cytoskeleton. Oncogene. 2008;27(23):3233–43. 10.1038/sj.onc.1210988 . 18071304

[25] Zhan Y, Modi N, Stewart AM, Hieronimus RI, Liu J, Gutmann DH, et al Regulation of mixed lineage kinase 3 is required for Neurofibromatosis-2-mediated growth suppression in human cancer. Oncogene. 2011;30(7):781–9. 10.1038/onc.2010.453 20890305 PMC3017676

[26] Laulajainen M, Melikova M, Muranen T, Carpen O, Gronholm M. Distinct overlapping sequences at the carboxy-terminus of merlin regulate its tumour suppressor and morphogenic activity. Journal of Cellular and Molecular Medicine. 2012;16(9):2161–75. Epub 2012/02/14. 10.1111/j.1582-4934.2012.01525.x . 22325036 PMC3822986

[27] Baser ME, Kuramoto L, Woods R, Joe H, Friedman JM, Wallace AJ, et al The location of constitutional neurofibromatosis 2 (NF2) splice site mutations is associated with the severity of NF2. Journal of medical genetics. 2005;42(7):540–6. 10.1136/jmg.2004.029504 15994874 PMC1736092

[28] Li W, Cooper J, Karajannis MA, Giancotti FG. Merlin: a tumour suppressor with functions at the cell cortex and in the nucleus. EMBO reports. 2012 10.1038/sj.embor.2012.11 PMC3323126 22482125

[29] Penney DP, Power JM, Frank M, Willis C, Churukian C. Analysis and testing of biological stains—the biological stain commission procedures. Biotech Histochem. 2002;77(5–6):237–75. 10.1080/bih.77.5-6.237.275 12564600

[30] WHO. WHO laboratory manual for the examination and processing of human semen 5th ed: Word Health Organization; 2010.

[31] Takubo K, Ohmura M, Azuma M, Nagamatsu G, Yamada W, Arai F, et al Stem cell defects in ATM-deficient undifferentiated spermatogonia through DNA damage-induced cell-cycle arrest. Cell Stem Cell. 2008;2(2):170–82. Epub 2008/03/29. 10.1016/j.stem.2007.10.023 S1934-5909(07)00240-8 [pii]. . 18371438

[32] Zhao B, Ye X, Yu J, Li L, Li W, Li S, et al TEAD mediates YAP-dependent gene induction and growth control. Genes Dev. 2008;22(14):1962–71. 10.1101/gad.1664408 18579750 PMC2492741

[33] Holm S. A simple sequentially rejective multiple test procedure. Scandinavian Journal of Statistics. 1979;6(2):65–70.

[34] Diniz MC, Pacheco ACL, Farias KM, de Oliveira DM. the eukaryotic flagellum makes the day—novel and unforeseen roles uncovered after post-genomics and proteomics data. Current Protein and Peptide Science. 2012;13:524–46. 22708495 10.2174/138920312803582951 PMC3499766

[35] Signorelli J, Diaz ES, Morales P. Kinases, phosphatases and proteases during sperm capacitation. Cell and tissue research. 2012;349(3):765–82. 10.1007/s00441-012-1370-3 . 22427115

[36] Jean-Faucher C, Berger M, de Turckheim M, Veyssiere G, Jean C. Developmental patterns of plasma and testicular testosterone in mice from birth to adulthood. Acta Endocrinologica (Copenh). 1978;89(4):780–8. Epub 1978/12/01. . 716781 10.1530/acta.0.0890780

[37] Martinez-Garcia F, Regadera J, Cobo P, Palacios J, Paniagua R, Nistal M. The apical mitochondria-rich cells of the mammalian epididymis. Andrologia. 1995;27(4):195–206. 7486029 10.1111/j.1439-0272.1995.tb01093.x

[38] Dacheux JL, Dacheux F. New insights into epididymal function in relation to sperm maturation. Reproduction. 2014;147(2):R27–42. 10.1530/REP-13-0420 . 24218627

[39] Primiani N, Gregory M, Dufresne J, Smith CE, Liu YL, Bartles JR, et al Microvillar size and espin expression in principal cells of the adult rat epididymis are regulated by androgens. Journal of andrology. 2007;28(5):659–69. 10.2164/jandrol.107.002634 . 17409466

[40] Crawley SW, Mooseker MS, Tyska MJ. Shaping the intestinal brush border. The Journal of cell biology. 2014;207(4):441–51. 10.1083/jcb.201407015 25422372 PMC4242837

[41] Crawford BD, Henry CA, Clason TA, Becker AL, Hille MB. Activity and distribution of paxillin, focal adhesion kinase, and cadherin indicate cooperative roles during zebrafish morphogenesis. Molecular biology of the cell. 2003;14:3065–81. 10.1091/mbc.E02- 12925747 PMC181551

[42] Giovannini M, Robanus-Maandag E, van der Valk M, Niwa-Kawakita M, Abramowski V, Goutebroze L, et al Conditional biallelic nf2 mutation in the mouse promotes manifestations of human neurofibromatosis type 2. Genes & Development. 2000;14:1617–30. 10.1101/gad.14.13.1617 10887156 PMC316733

[43] Hilton DA, Hanemann CO. Schwannomas and their pathogenesis. Brain pathology. 2014;24(3):205–20. 10.1111/bpa.12125 . 24450866 PMC8029073

[44] Menges CW, Kadariya Y, Altomare D, Talarchek J, Neumann-Domer E, Wu Y, et al Tumor suppressor alterations cooperate to drive aggressive mesotheliomas with enriched cancer stem cells via a p53-miR-34a-c-Met axis. Cancer Res. 2014;74(4):1261–71. 10.1158/0008-5472.CAN-13-2062 24371224 PMC3945416

[45] Yang S, Weng H, Chen L, Guo X, Parra M, Conboy J, et al Lack of protein 4.1G causes altered expression and localization of the cell adhesion molecule nectin-like 4 in testis and can cause male infertility. Molecular and Cellular Biology. 2011;31(11):2276–86. 10.1128/MCB.01105-10 21482674 PMC3133242

[46] Dorogova NV, Akhmametyeva EM, Kopyl SA, Gubanova NV, Yudina OS, Omelyanchuk LV, et al The role of Drosophila Merlin in spermatogenesis. BMC Cellular Biology. 2008;9:1 10.1186/1471-2121-9-1 18186933 PMC2253521

[47] Bolobolova EU, Yudina OS, Dorogova NV. Drosophila tumor suppressor merlin is essential for mitochondria morphogenesis during spermatogenesis in Drosophila melanogaster. Cell and Tissue Biology. 2011;5(2):136–43. 10.1134/s1990519x11020040

[48] Rossi P, Dolci S, Sette C, Capolunghi F, Pellegrini M, Loiarro M, et al Analysis of the gene expression profile of mouse male meiotic germ cells. Gene Expression Patterns. 2004;4(3):267–81. 10.1016/j.modgep.2003.11.003 . 15053975

[49] Xu H, Gutmann DH. merlin differentially associates with the microtubule and actin cytoskeleton. Journal of Neuroscience Research. 1998;51:403–15. 9486775 10.1002/(SICI)1097-4547(19980201)51:3<403::AID-JNR13>3.0.CO;2-7

[50] Fernandez-Valle C, Tang Y, Ricard J, Rodenas-Ruano A, Taylor A, Hackler E, et al Paxillin binds schwannomin and regulates its density-dependent localization and effect on cell morphology. Nat Genet. 2002;31(4):354–62. 10.1038/ng930 . 12118253

[51] Grönholm M, Sainio M, Zhao F, Heiska L, Vaheri A, Carpen O. homotypic and heterotypic interaction of the neurofibromatosis 2 tumor suppressor protein merlin and the ERM protein ezrin. Journal of cell science. 1999;112:895–904. 10036239 10.1242/jcs.112.6.895

[52] Scoles DR, Huynh DP, Morcos PA, Coulsell ER, Robinson NG, Tamanoi F, et al Neurofibromatosis 2 tumour suppressor schwannomin interacts with betaII-spectrin. Nature Genetics. 1998;18(4):354–9. Epub 1998/04/16. 10.1038/ng0498-354 . 9537418

[53] Wong EW, Mruk DD, Cheng CY. Biology and regulation of ectoplasmic specialization, an atypical adherens junction type, in the testis. Biochimica et biophysica acta. 2008;1778(3):692–708. 10.1016/j.bbamem.2007.11.006 18068662 PMC2701193

[54] Gungor-Ordueri NE, Tang EI, Celik-Ozenci C, Cheng CY. Ezrin is an actin binding protein that regulates sertoli cell and spermatid adhesion during spermatogenesis. Endocrinology. 2014;155(10):3981–95. 10.1210/en.2014-1163 25051438 PMC4164919

[55] Itman C, Bielanowicz A, Goh H, Lee Q, Fulcher AJ, Moody SC, et al Murine inhibin alpha subunit haploinsufficiency causes transient abnormalities in prepubertal testis development followed by adult testicular decline. Endocrinology. 2015:en20141555 10.1210/en.2014-1555 . 25781564

[56] Hess RA. Effects of environmental toxicants on the efferent ducts, epididymis and fertility. J Reprod Fertil Suppl. 1998;53:247–59. 10645284

