# Supplementary figures and images for: Merlin Isoforms 1 and 2 Both Act as Tumour Suppressors and Are Required for Optimal Sperm Maturation
Source: PLoS One. 2015 Aug 10;10(8):e0129151. doi: 10.1371/journal.pone.0129151 (PMC4530865; doi:10.1371/journal.pone.0129151)

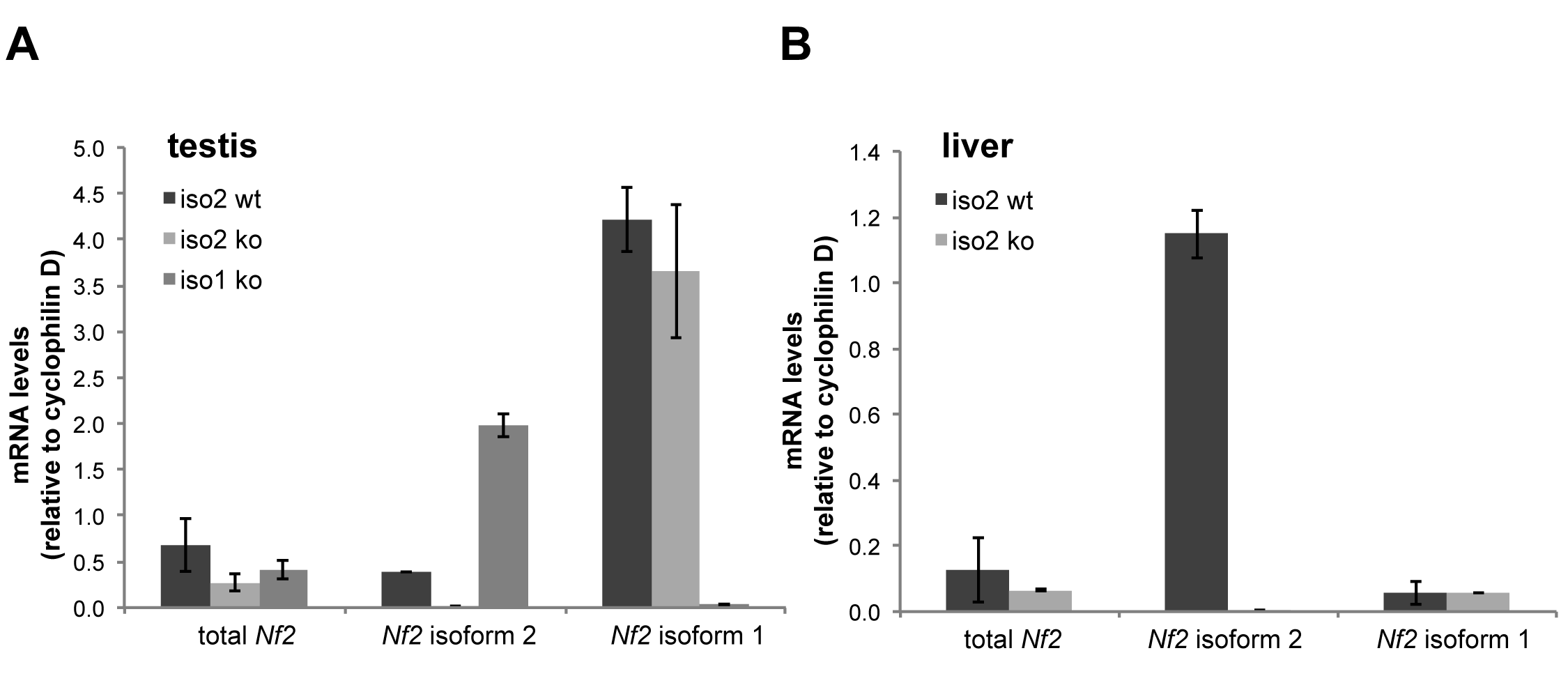

Supplement: S1 Fig — (A) qPCR analysis of Nf2 expression in testes demonstrating the specificity of the isoform primers used. Deletion of isoform 1 clearly increased isoform 2 mRNA levels in the testis (n = 2 for each genotype). (B) qPCR analysis of iso2 ko liver Nf2 mRNA showed successful deletion of isoform 2 mRNA, but in contrast to the clear induction of isoform 1 protein levels, mRNA levels remained similar to the wildtype (n = 3 for each genotype). Note that isoform 1 and 2 do not add up to total Nf2 levels due to the use of a dT18 primer, lessening the amount of cDNA containing the 5’ mRNA region. In all cases the total level of Nf2 mRNA was reduced compared to the wildtype. However, Merlin protein levels did not correspond clearly to the mRNA levels (Fig 6D and 6E, S3 Fig). (TIF) [file pone.0129151.s001.tif]

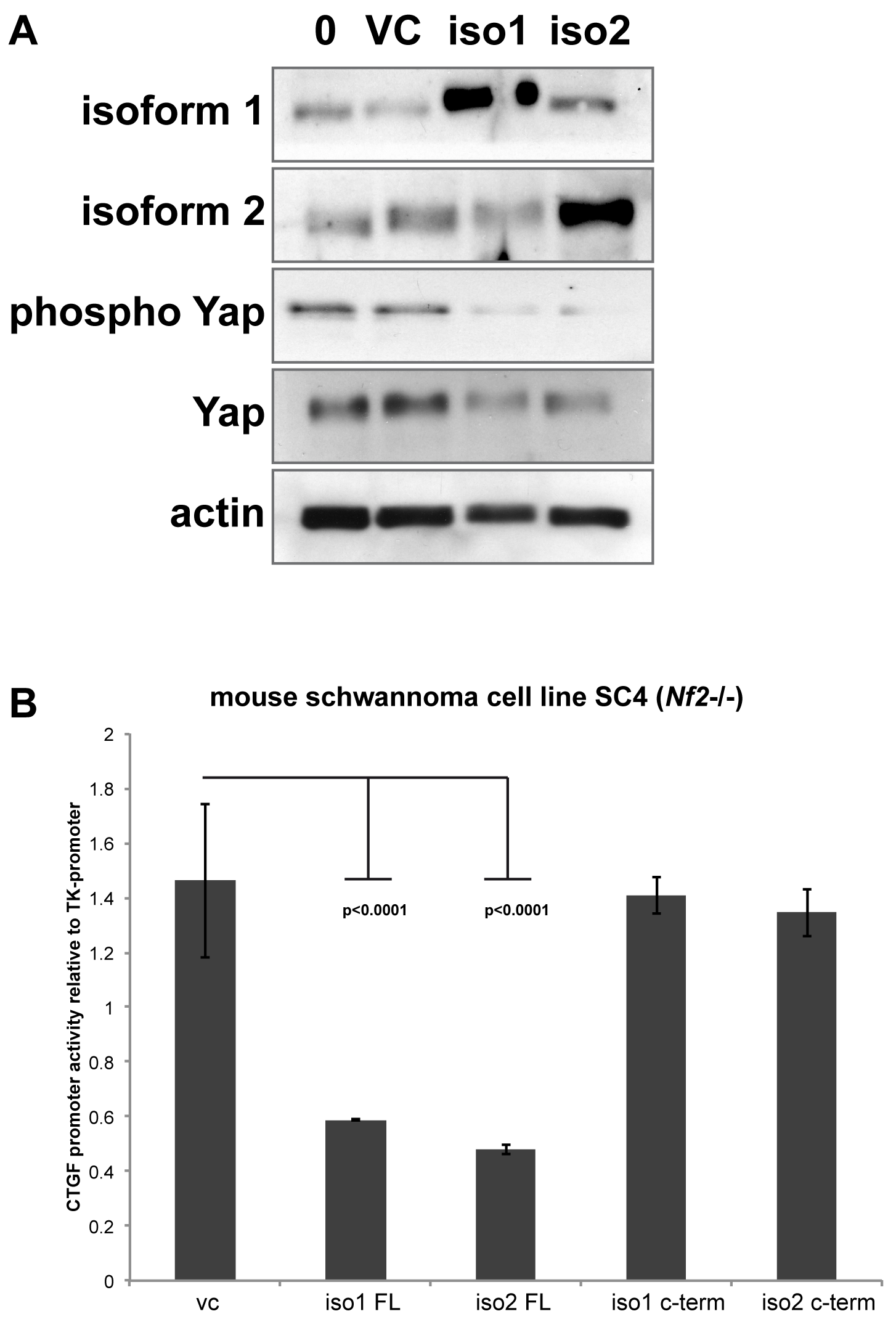

Supplement: S2 Fig — (A) Expression of isoform 1 or 2 decreased Yap stability in the human hepatoma cell line C3A. Western blot analysis using isoform specific antibodies generously supplied by D. Gutmann, described in Schulz et al. 2013. (B) Expression of either isoform suppressed activity of the YAP-driven CTGF promoter in Nf2-/- SC4 schwannoma cells indicating Hippo pathway activation by both merlin isoforms. Firefly luciferase reporter assays normalized to TK-promoter driven luciferase activity. (0: non-transfected control, VC: vector control, FL: full length protein, c-term: carboxy terminal half of Merlin. (TIF) [file pone.0129151.s002.tif]

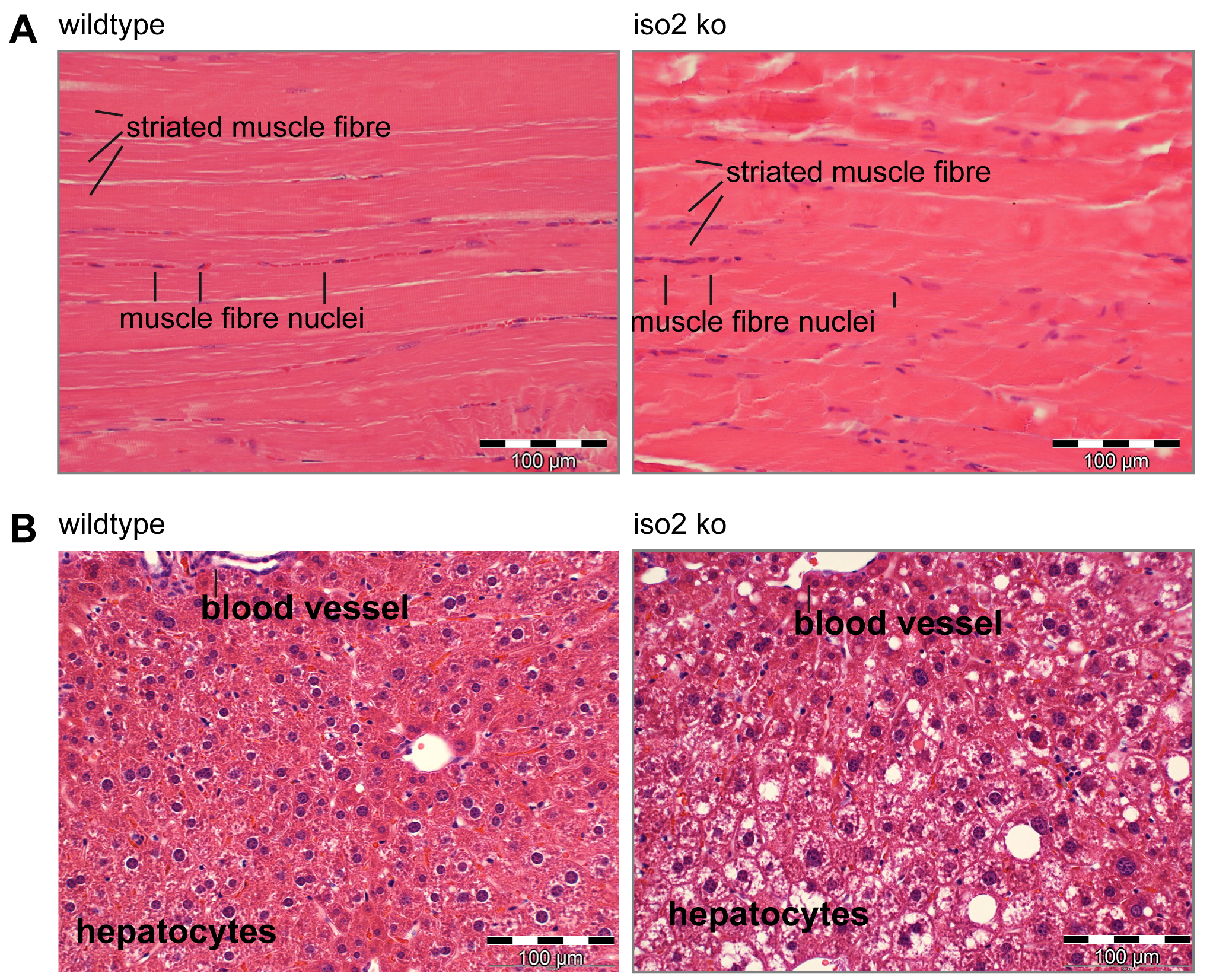

Supplement: S3 Fig — H&E stained histological sections of skeletal muscle (A) and liver (B) of adult (3–6 months) old animals. No pathological changes were observed. (scale bars = 100 μm) (TIF) [file pone.0129151.s003.tif]

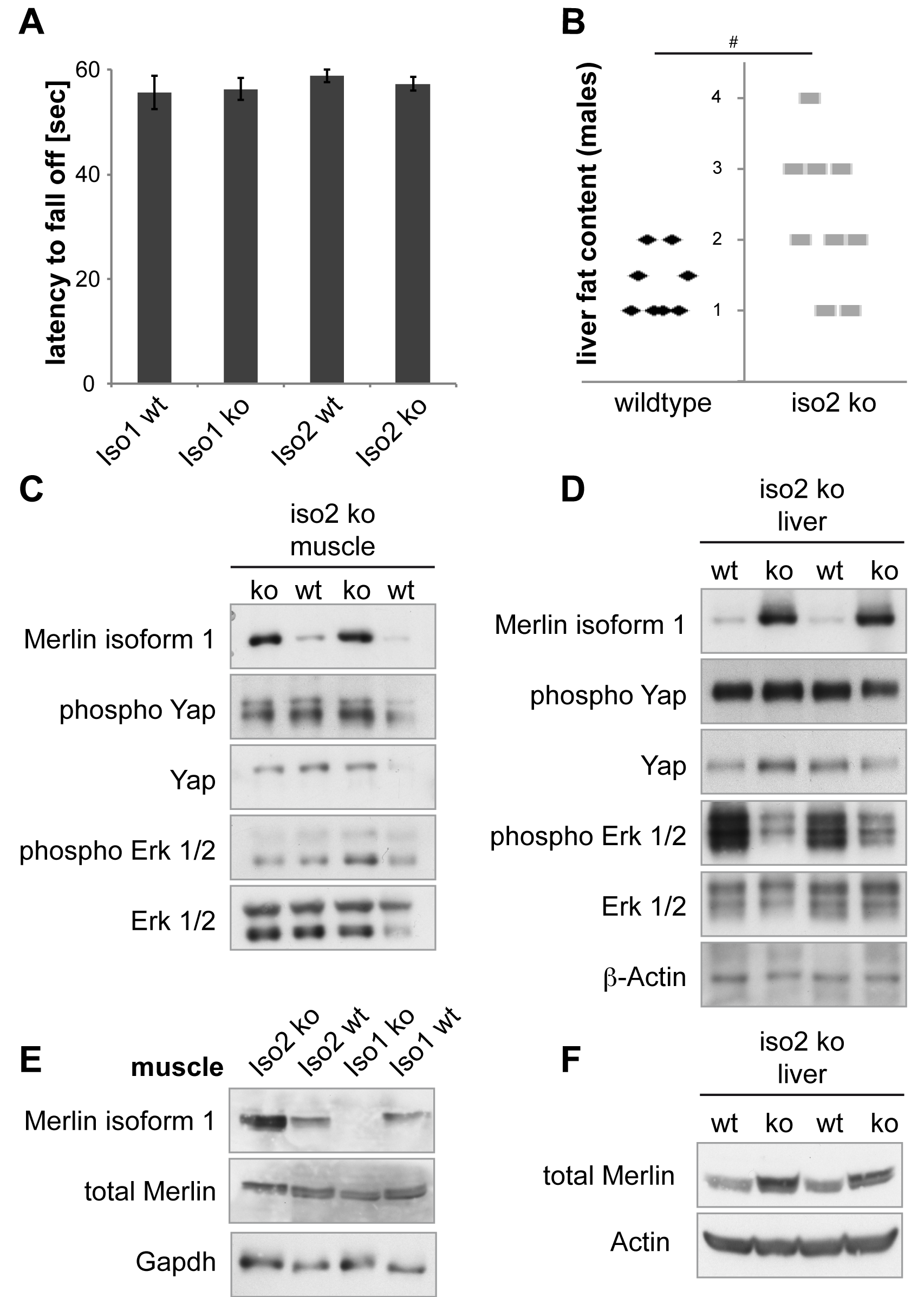

Supplement: S4 Fig — (A) Muscle strength as assessed in a hanging wire test was not affected by either knockout (n = 4 for each genotype). (B) Liver fat content was visualized by Oil Red O staining and categorized independently by AZ and SM. The median liver fat content of male iso ko mice was slightly increased (Mann-Whitney U-test, #: significance level α<0.10, n(wt) = 8, n(iso2 ko) = 9). (C) Western blot analysis of iso2 ko muscle showed similar Hippo (phospho Yap) and MAPK (phospho Erk) activity in knockout and wildtype. (D) Loss of isoform 2 in liver decreased Erk phosphorylation in Western blot analysis. Western blot analysis of muscle tissue (E) confirmed similar total merlin levels in both knockouts, whereas the level of total Merlin was upregulated in iso2 ko liver (F). (TIF) [file pone.0129151.s004.tif]
